# The role of the family in youth psychiatric readmission: a scoping review

**DOI:** 10.1007/s00787-026-03062-y

**Published:** 2026-06-06

**Authors:** Amos-Silvio Erik Friedrich, Magdalena Siegel, Joy Busch, Madlene Radosavljevic, Guy Bodenmann, Martina Zemp

**Affiliations:** 1 Department of Clinical and Health Psychology, University of Vienna, Vienna, Austria; 2 Department of Psychology, University of Zurich, Zurich, Switzerland

**Keywords:** Readmission, Psychiatry, Family, Youth, Scoping review

## Abstract

Repeated admissions of children and adolescents after inpatient psychiatric treatment point to underrecognized issues in the post-discharge period. The family environment is a promising target for sustained recovery. However, recent systematic reviews on predictors of psychiatric readmission in children and adolescents were unable to synthesize most family-level factors due to conceptual and methodological differences. To address this, we conducted a scoping review following PRISMA-ScR guidelines to (a) synthesize literature on family-related structural and process variables associated with youth psychiatric readmission and (b) identify conceptual and methodological gaps. We included primary and secondary studies in English or German language examining family-related constructs in relation to inpatient readmission. Eighty-three reports retrieved via a multi-tiered search strategy (including PubMed, PsycInfo, Scopus, Web of Science, ProQuest Dissertations & Theses Global, and Google Scholar) were included (79 empirical studies, three study protocols, and one meta-analytic review). Empirical studies have increased in recent years, were primarily conducted in the United States, and largely relied on retrospective or record-based data. Evidence was concentrated on structural characteristics. Among the examined constructs, results linked readmission to indicators of structurally burdened or disrupted caregiving environments (e.g., institutional or foster care), family psychiatric problems (e.g., history of or current psychopathology), and treatment involvement. In contrast, research on specific family processes remains sparse despite their established significance in adult populations. Findings support the relevance of various family factors for youth psychiatric readmission but highlight gaps regarding complex associations, short-term risks, and prospective studies of family processes.

**Supplementary Information:**

The online version contains supplementary material available at https://doi.org/10.1007/s00787-026-03062-y.

## Introduction

Up to one third of children and adolescents who receive inpatient psychiatric treatment are readmitted within one year, most within the first three months [1–3]. Repeated inpatient admissions impose substantial costs on healthcare and social systems and can cause severe psychological distress for young patients and their families [4, 5]. While research on readmission has largely focused on clinical and treatment characteristics (for an overview, see [6]), the shift toward early outpatient and community-based care has made the post-discharge period increasingly important for prognostic outcomes [7, 8]. In this context, the family environment to which children and adolescents return gains a central role in maintaining improvements. While a recent review [9] has highlighted family functioning as a key target, it has also identified a knowledge gap in how families contribute to sustained mental health recovery after youth’s inpatient psychiatric stay. Therefore, the main goal of this scoping review is to map out what is already known and what is still needed in future research on the role of the family for youth psychiatric readmission (i.e., family factors associated with readmission, as well as family-based interventions aimed at preventing readmission).

Readmission is a commonly used indicator of service utilization often interpreted in relation to care quality and symptomatic relapse. Although generally viewed as an adverse outcome, it can also represent an appropriate and necessary component of care in cases of acute crises or when outpatient resources are insufficient. Readmission must therefore be understood within the broader care system in which it occurs. This complexity is reflected in theoretical frameworks such as the Network Episode Model (NEM) [10] which conceptualizes service utilization as shaped by the interaction of formal care, community and family network factors. Consistent with this view, previous research shows that readmission is influenced by access to services and resources [11] as well as psychosocial factors [1, 12]. Adapting the NEM to youth mental health service use, Costello et al. [13] highlight caregivers’ gatekeeping role in their Family Network-Based Model. This model posits that caregiver knowledge, resources, and beliefs around where and when to seek professional help considerably shape youth mental health pathways beyond symptom severity. It further argues that for minor patients, many patient-level characteristics in adult models (such as income, residential location, or insurance status) are more appropriately considered as family-level characteristics [13]. As such, family-related variables constitute hybrid indicators that span proximal family context as well as more distal socioeconomic and administrative conditions. Accordingly, family influences on (inpatient) service use can be distinguished into direct effects on utilization and indirect effects on youth mental health [14], requiring attention to both structural and process-related dimensions.

A useful framework of family processes is presented in the Triadic Model of Family Process [15]. According to this model, family processes affecting child psychopathology can be conceptualized at three levels: (i) at the level of processes involving the family as a whole (i.e., *family-level processes*, e.g., family functioning, family support); (ii) at the level of parental characteristics (i.e., *parent-level processes*, e.g., parental mental health, interparental relationship quality); and (iii) within dyadic relationships involving the child (i.e., *dyad-level processes*, e.g., parenting behavior, sibling relationship quality). In addition, broader structural family characteristics, such as low socioeconomic status [16], caregiving arrangement [17], or minority status [18] can affect family processes at all three levels and subsequently child adjustment by the associated distress, as proposed by family stress models [16, 19]. In contrast to the vast literature on the impact of family processes on general youth health outcomes [e.g., 20–22], relatively little attention has been devoted to their specific role in the context of psychiatric readmission [23]. For the current review, we adopt the triadic framework outlined above [15], extended by a structural level, and propose that factors affecting readmission can be identified at each of these levels.

For child and adolescent psychiatry, literature generally emphasizes the meaning of family support in transitioning out of hospital care [7, 24], and studies of affected children and adolescents and their caregivers underscore the importance of a supportive family environment after discharge [25–27]. Recent family-focused intervention studies have also shown reductions in readmission rates [28, 29]. Nevertheless, scholars stress the need for a better understanding of effective family aftercare due to the limited evidence base on family factors affecting readmissions [7, 23, 29]. This gap is exacerbated by a lack of comparability across the available studies: Two recent systematic reviews on various predictors of youth readmission [1, 23] were unable to synthesize most family-related variables due to the high conceptual heterogeneity in methods and measures. However, these reviews either focused on meta-analytic synthesis and did not elaborate on results excluded from these quantitative analyses [1], or were limited to studies from the US and thus restricted in their generalizability to the healthcare systems of other countries and cultures [23]. In addition, both reviews included studies up to 2018, which means that the recent developments in this growing field have not yet been considered. Moreover, while proven to be especially important in the context of mental health, social determinants of health and healthcare use are only starting to be assessed comprehensively, prospectively, and systematically [30]. Readmission as an outcome is most often examined using large-scale record data, which commonly lack information on social context due to inherent shortcomings of the data source [31]. Hence, when studying family factors and interventions in the context of youth psychiatric readmission, it is also necessary to consider the methodological challenges raised by limitations of available data sources.

Taken together, a broad, systematic approach to review and synthesize relevant literature on family-related factors associated with readmission of children and adolescents who have previously received inpatient psychiatric treatment is needed. To this end, given the relatively sparse current state of research and the methodological issues and incompatibilities across studies, we have opted for the methodology of a scoping review. This approach is particularly well suited to map the breadth and nature of evidence in complex and heterogeneous fields [32].

## Current study

In this scoping review, we aimed to systematically collate existing literature on the role of family factors related to readmission in child and adolescent psychiatry to provide an overview of what is known from previous research in this field. Specifically, the research questions (RQs) guiding our review were the following:

- RQ 1: What is known about the role of the family for psychiatric readmission of children and adolescents who have previously received inpatient psychiatric treatment?
  - What family-related structural and process variables (mapped onto the family, parent, and dyad level) are associated with psychiatric readmission of children and adolescents?
  - How do findings relate to and fit into the broader research and theories on family systems and processes affecting children and adolescents’ mental health?
- RQ 2: How has research on the role of the family for psychiatric readmission of children and adolescents been conducted?
  - What methods and sources of evidence (e.g., qualitative/quantitative, prospective/retrospective, primary/secondary data, country of study) have been used to examine family-related structural and process variables in the context of child and adolescent psychiatric readmission?
  - What recommendations have been made in the last decade for conceptual and methodological innovation in future research?

We examined these research questions to build upon previous reviews by explicitly and exclusively focusing on family-related constructs and applying a scoping review methodology [33] equipped to systematically synthesize conceptually and methodologically heterogenous reports. Given the recent upsurge of research on youth psychiatric readmission, this scoping review also provides a synthesis of the latest developments in the field. We contextualize and structure our findings along the triadic family process framework and identify gaps in the current literature to derive directions for future research.

## Method

### Protocol preregistration and open science practice adherence

This scoping review adheres to PRISMA-ScR guidelines for scoping reviews [33], the PRISMA-S [34] guidelines for systematic literature searches, as well as the PRISMA [35] guidelines for systematic reviews where preferable due to their recency. We preregistered our study protocol prior to the systematic literature search on the Open Science Framework (OSF) on January 5, 2023, and issued an amendment on August 10, 2023, entailing an updated selection of included databases, changes to one data extraction item (gender composition), and the exclusion of case reports. The original and amended versions of the study protocol, all study materials and data to reproduce all results, figures, and tables within the main text, and supplementary materials are available via https://osf.io/qfzew.

### Eligibility criteria

We defined eligibility criteria following the (S)PCC framework (study types, population, concept, context) recommended for scoping reviews [32].

#### Study types

We included original quantitative, qualitative, or mixed methods studies as well as systematic research syntheses, such as systematic reviews with or without meta-analyses reported in peer-reviewed journals, book chapters, research reports, or dissertations, as well as other grey literature sources (all these study types are henceforth collectively referred to as ‘studies’). We excluded conference abstracts, qualification theses at bachelor and master level (i.e., undergraduate or graduate level below the doctoral degree), and non-empirical, conceptual works. Articles had to be written in English or German to be eligible for inclusion. No restrictions on the year of publication or data collection were applied.

#### Population

Studies were included if they reported on a sample or separate subsample of individuals under 20 years of age at the time of first data collection (i.e., upper bound of age range below 20;0 years or, in case no such data was retrievable, sum of sample mean and standard deviation below 20;0 years to ensure a majority of participants are within the age range) who underwent inpatient treatment (defined as at least one overnight stay) at mental health facilities prior to or during the course of the study. We chose to focus on individuals below 20 years of age in accordance with the WHO definition of adolescence between ages 10 to 19 [36]. However, we also included studies on adults above the specified age range at the time of data collection when they included a retrospective assessment of the relevant age period (i.e., participants were under 20 years). We use the term *youth* to refer to individuals within the defined age range when no more specific designation applies.

#### Concept

Studies were eligible for inclusion if they either implemented family-based interventions or examined family-related constructs. The examined or targeted constructs could include (1) structural variables characterizing the family or caregiving context (e.g., caregiving arrangement, family form), including indicators of family socioeconomic status (e.g., family income, insurance type) [13, 37] and minority status [18] as well as (2) process variables at the three levels according to the Triadic Model of Family Process by Schleider and Weisz [15]: (2a) the family level (e.g., family functioning, family climate, family support), (2b) the parent level (e.g., parental psychopathology, caregiver burden, interparental relationship quality), and (2c) the dyad level involving the child (e.g., expressed emotion, parenting behavior, maltreatment, parent-child conflict, sibling relationship quality). Any mode of assessment for family constructs was deemed eligible (i.e., self-report or proxy-report [e.g., by parents, clinicians] questionnaires or rating scales, live/recorded observation, and/or interviews).

#### Context

To be eligible for inclusion, studies needed to have examined any of the above specified family-related constructs or interventions in relation to children and adolescents’ repeated inpatient admission (i.e., readmission) to a mental health facility (the same as previously or any other). Following common research practice [38–40], we defined readmission as at least one night in inpatient care at any point after discharge from prior inpatient treatment. Assessed indicators of readmission needed to be the rate of readmission in a specified timeframe [41] or the time to readmission [42] following discharge and could have been assessed by self-report, proxy-report (e.g., by parents, clinicians), or health record data.

### Search strategy and information sources

We employed a multi-tiered search strategy relying on database searches, cited reference searches, publication lists of relevant authors in the field, and study requests posted to relevant listservs. The full search strategy and its development are outlined in detail in the study protocol. For the systematic database search, we searched titles, abstracts, and keywords of records in the following databases on January 17, 2023, from their respective dates of inception: *PubMed*,* PsycInfo* (via EBSCOhost), *Scopus*,* Web of Science* (Core Collection as subscribed to by the University of Vienna), *ProQuest Dissertations & Theses Global*. In addition, we considered *Google Scholar* results (first 200 hits) for selected terms related to youth psychiatric readmission (see protocol) and searched preregistrations at *ClinicalTrials* (via clinicaltrials.gov, condition “psychiatric hospitalization” and filtered by “child”). Our search strings combined four sets of keywords developed along our (S)PCC criteria related to (1) readmission/rehospitalization (e.g., readmission*, repeat* inpatient), (2) psychiatry/mental health (e.g., mental, psych*), (3) youth (e.g., child*, adolescen*), and (4) family (e.g., famil*, parent*), which we combined using the AND operator. The full search strings for each database are available in the study protocol and were used as preregistered.

To ensure that the search would include the most recent developments in the field, the initial database search was updated on August 5, 2024, and September 2, 2025. For each update, we repeated queries in all databases and assessed any records added after the previous search for eligibility.

### Selection process

Records were deduplicated in Endnote following the steps described by Bramer et al. [43]. Deduplicated records were screened in parallel by the first and third author. The selection process and agreement statistics are detailed in Online Resource S1.

### Data charting process

Information on all data items (see below) was extracted in duplicate and independently by the first (AF) and either the third (JB, original search) or fourth author (MR, updated searches). We used a standardized spreadsheet piloted (AF, JB) on a subset of three eligible studies (see OSF). All discrepancies were resolved through discussion within the coders or with another author (MZ), therefore, we did not calculate category-level agreement.

As is customary when conducting scoping reviews [32, 33], we did not conduct a systematic assessment of the methodological quality or risk of bias of the included studies, as our focus was on the descriptive summary of existing research with particular attention to current gaps in the literature. However, as part of our research aim, we coded study limitations as reported by study authors to give readers an impression of the study’s main limitations.

### Data items

From each study eligible for inclusion, we extracted information on publication and study characteristics (year and authors, methods and design), sample characteristics (population, composition), and conceptual information (aims, examined constructs or intervention, findings, limitations and recommendations). Extracted items are detailed in Online Resource S1 and our coding sheet on OSF.

Qualitative results from qualitative and mixed-methods studies relating aspects of the family environment to readmission were summarized by the review authors. For quantitative study results, we further extracted data on individual associations. These included variable, type (interventional/non-interventional), family level (structural, family, parent, dyad), timeframe considered for readmissions (e.g., 30, 180, 365 days), meaningful association (yes/no, see definition below), effect (elevated readmission coded as risk, lowered readmission coded as protective, otherwise coded as no effect), and covariates (yes/no). We considered interventional results to be any results related to variables describing family-based interventions, including controlled or pre-post comparisons, reports of received support, or observational designs. Note that both interventional and non-interventional results could be reported within the same study. For quantitative results, we coded associations as meaningful if they proved statistically significant at α = 0.05 or had a non-trivial effect size corresponding to *r* ≥ .10 in bivariate (preferred) or equivalent multivariate (extracted only if no bivariate association was reported) analyses. This approach was chosen with the goal of mapping substantively relevant associations. The threshold was set following guidelines describing meaningful effects starting at *r* = .1 for highly valid [44] and clinically consequential outcome measures in the context of longer-term population outcomes [45].

### Synthesis of results

The synthesis of results was guided by our research questions outlined above. In addition to narrative and tabular presentation, we employed visualization (geographic maps, bubble charts, timelines, bar plots), where possible, as recommended for innovative and clear synthesis of scoping reviews [46, 47].

RQ 1 was primarily examined at the level of individual study results (RQ 1a). For quantitative study results, we used all extracted results. Consequently, the summary includes multiple results for the same construct from studies that examined different timeframes (e.g., 30-day readmissions and 180-day readmissions) or reported separate associations for multiple comparison categories (e.g., institutional care and adoptive parents compared with biological parents). Results are presented across bivariate and multivariate associations as separate comparison did not show a distinct pattern (see Online Resource S3). Interventional results were grouped by the type of intervention and stratified by the assessed timeframes. Non-interventional associations were grouped by construct and stratified by timeframe and family level (i.e., structural, family, parent, dyad). For each categorized construct, we present a ratio of meaningful to total associations (m/t; to be read as: of a total of t reported associations, m were meaningful according to our threshold).

Results from qualitative and mixed-methods studies were extracted from their results and discussion sections and synthesized narratively. Review results regarding family variables were summarized in text. Protocols were described in terms of their study aims, location, and design. We address how findings from these studies relate to broader family psychology research (RQ 1b) within our discussion of this review’s results.

Data extracted on the level of complete studies (i.e., publication, study, and sample characteristics as well as conceptual information) was examined to address RQ 2. Methods and sources of evidence (RQ 2a) were charted to present frequencies of combinations by study type. We extracted excerpts on current limitations and recommendations for future research (RQ 2b) within the last decade from the included full texts and grouped them into overarching themes across records.

## Results

### Selection of sources of evidence

**Fig. 1:**
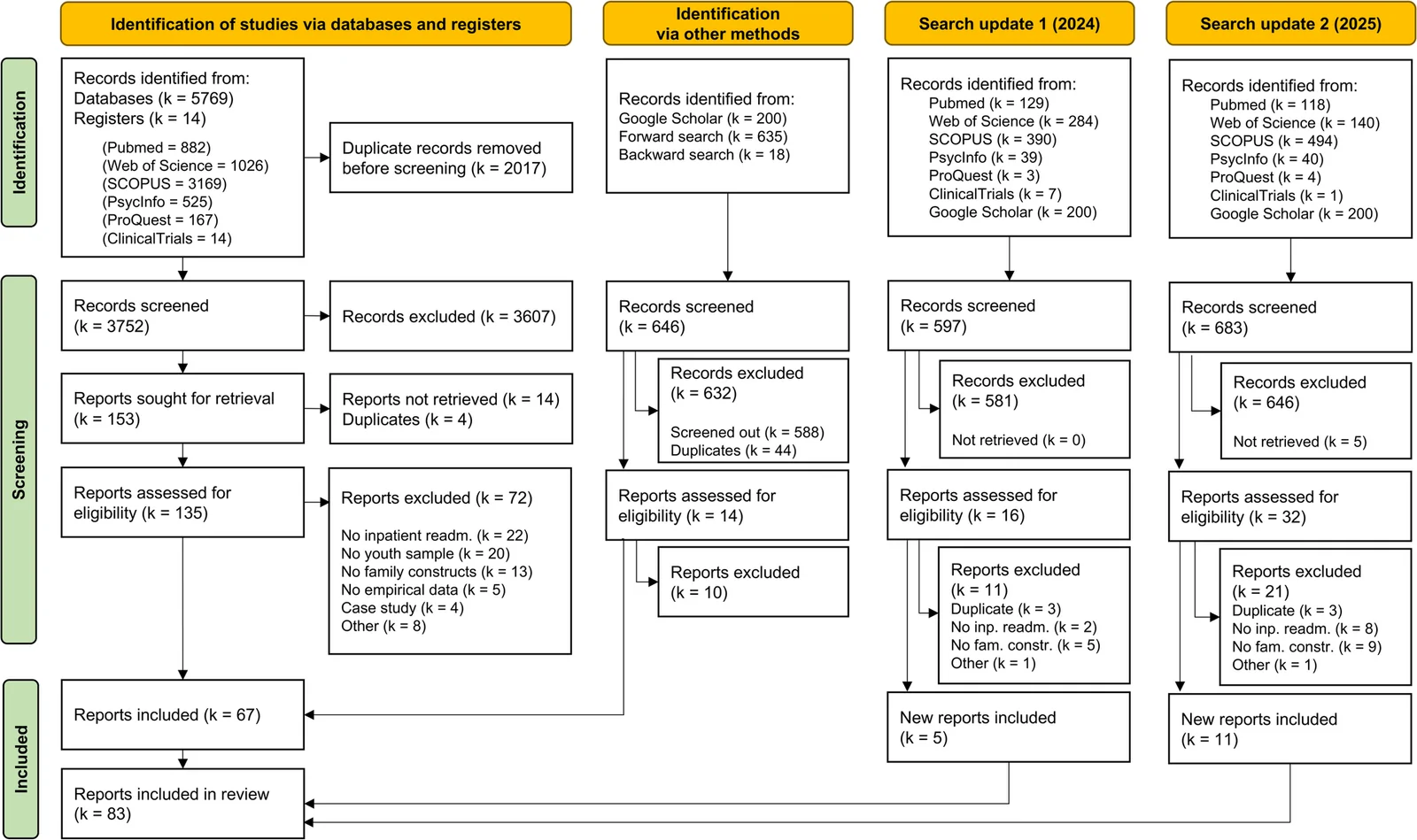
PRISMA flowchart for the original and the updated searches

The selection process is detailed in the PRISMA flow chart (see Fig. 1). The initial search yielded *k* = 3752 records (after deduplication) from systematic database searches. Of the records sought for retrieval (*k* = 153), after discarding non-retrieved records, the remaining sources (*k* = 135) were then independently assessed by the same two authors (AF, JB). An additional *k* = 646 records were identified via other methods and assessed for eligibility. Full text assessment yielded *k* = 67 included reports. Across both updates, *k* = 16 new reports were identified, resulting in a total of *k* = 83 reports included in this review.

### Characteristics of sources of evidence

**Fig. 2:**
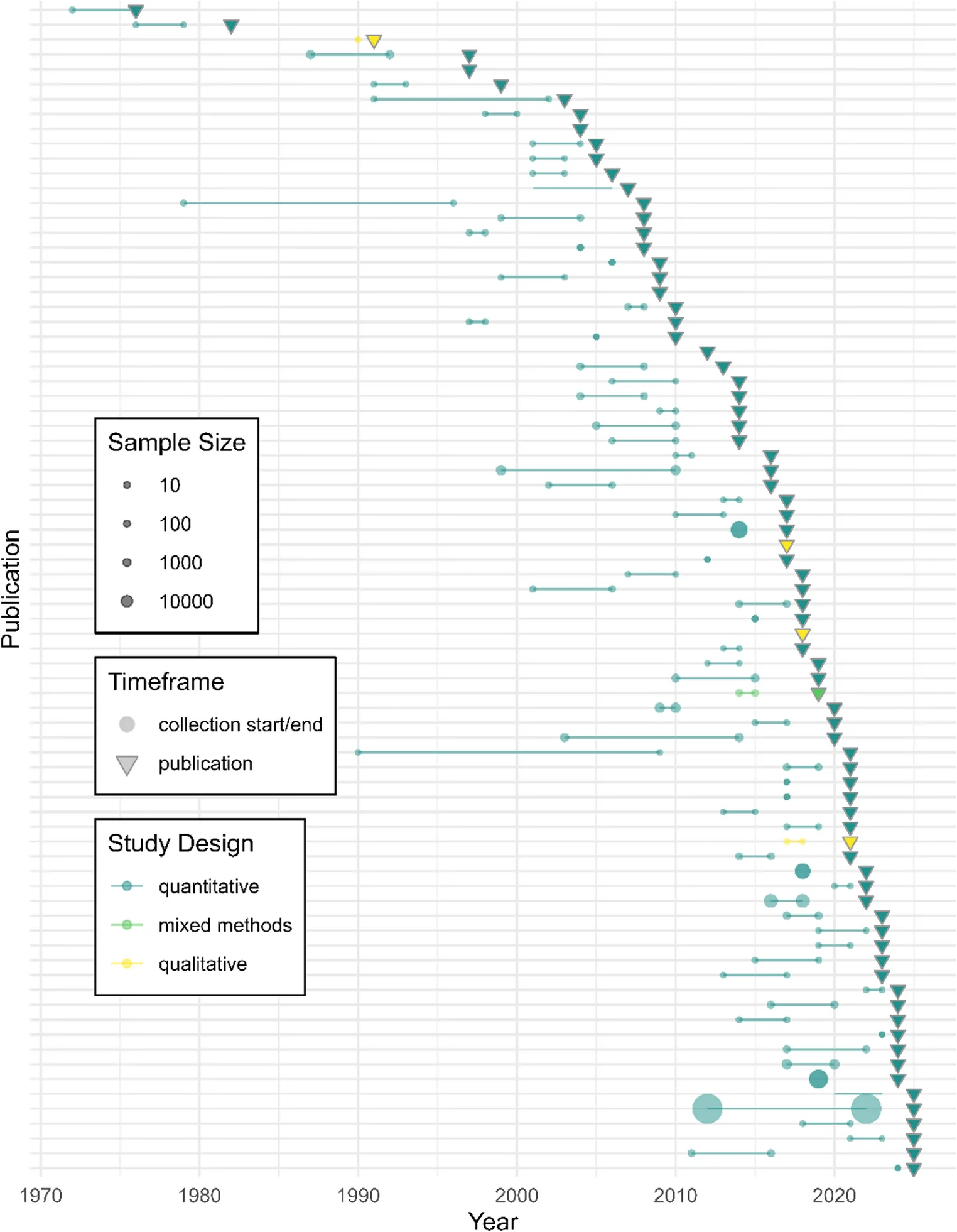
Overview of included studies by date, sample size, and study type

**Fig. 3:**
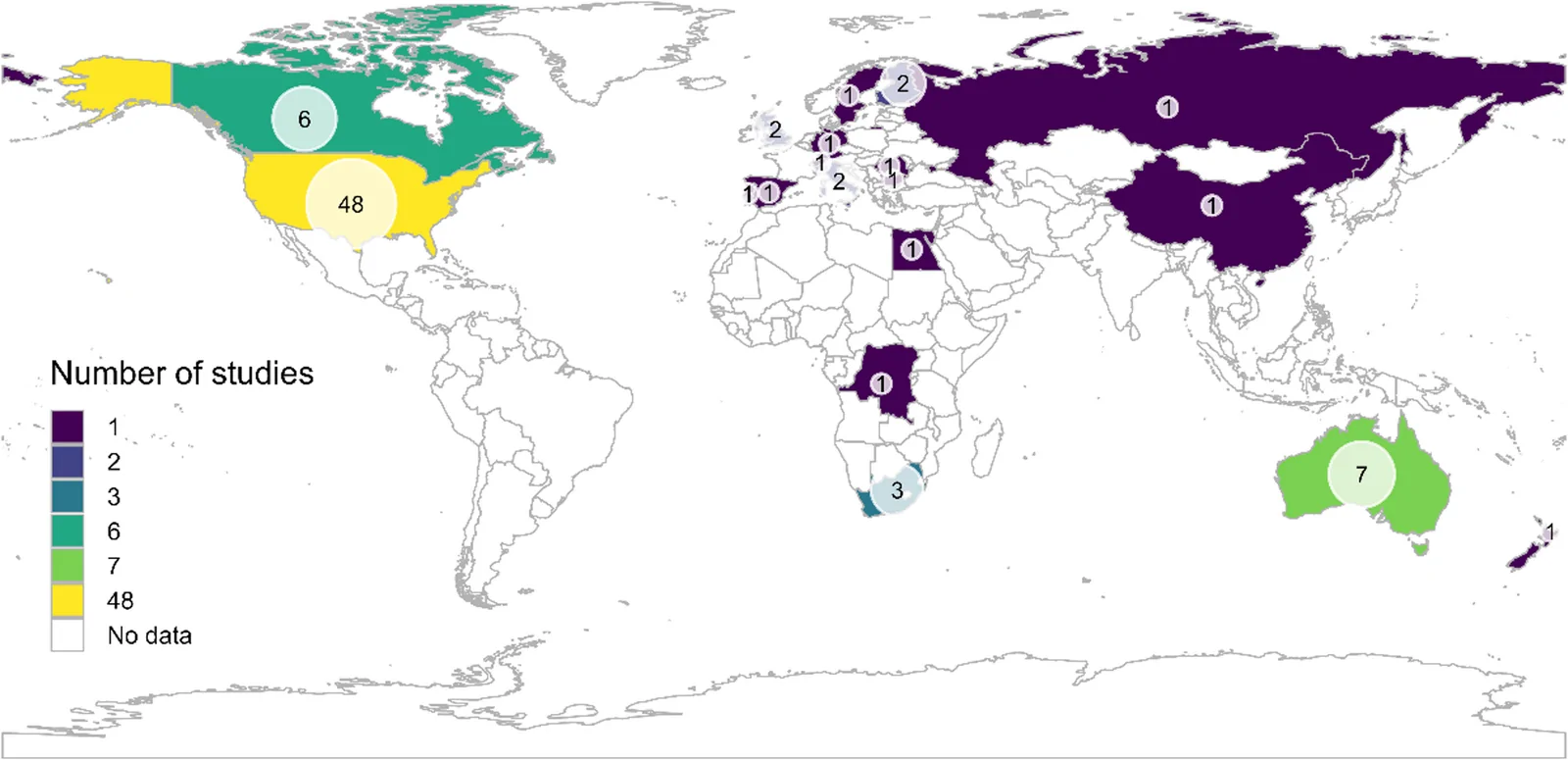
Distribution of included studies by country. Note. Countries of review and protocol records are excluded. One study (ID 64) collected data in multiple countries and was counted once for each country

Details on the characteristics of all included reports (79 empirical studies, 3 study protocols, 1 review) can be found in Online Resource S2. Figure 2 provides an overview of the *k* = 79 included empirical studies in terms of sample size, study design, and timeframe. These were published between 1976 and 2025 and examined timespans up to 19 years. Most of them were published as journal articles (*k* = 72, 91.1%), with a small number of studies reported in dissertation theses (*k* = 7, 8.9%). The number of publications has increased in recent years, starting around 2010. Most studies employed quantitative designs (*k* = 74, 93.7%), with the remainder being qualitative (*k* = 4, 5.1%) and mixed-methods studies (*k* = 1, 1.1%). Sample sizes ranged widely from 7 to 239,541 (median sample size *n* = 224). As Fig. 3 depicts, almost two thirds of studies were conducted in the United States (*k* = 48, 60.8%), Australia (*k* = 7, 8.9%), and Canada (*k* = 6, 7.6%). Other countries represented were South Africa (*k* = 3, 3.8%), Finland (*k* = 2, 2.5%), Italy (*k* = 2, 3.5%), the United Kingdom (*k* = 2, 2.5%), and several countries where only one study was conducted (*k* = 9, 11.4%).

Apart from primary empirical studies, we included one meta-analytic systematic review comprising 33 primary studies covering study timeframes between 1997 and 2018 [1] and three protocols. These protocols covered a mixed-methods study and a randomized-controlled trial conducted in Germany (IDs P2, P3) and a randomized-controlled trial in the US (ID P1). Data collection periods outlined in the protocols concluded in 2021 (IDs P1, P2) and 2025 (ID P3).

### RQ 1: The role of the family for psychiatric readmission of children and adolescents

#### Findings from quantitative studies: noninterventional results

**Fig. 4:**
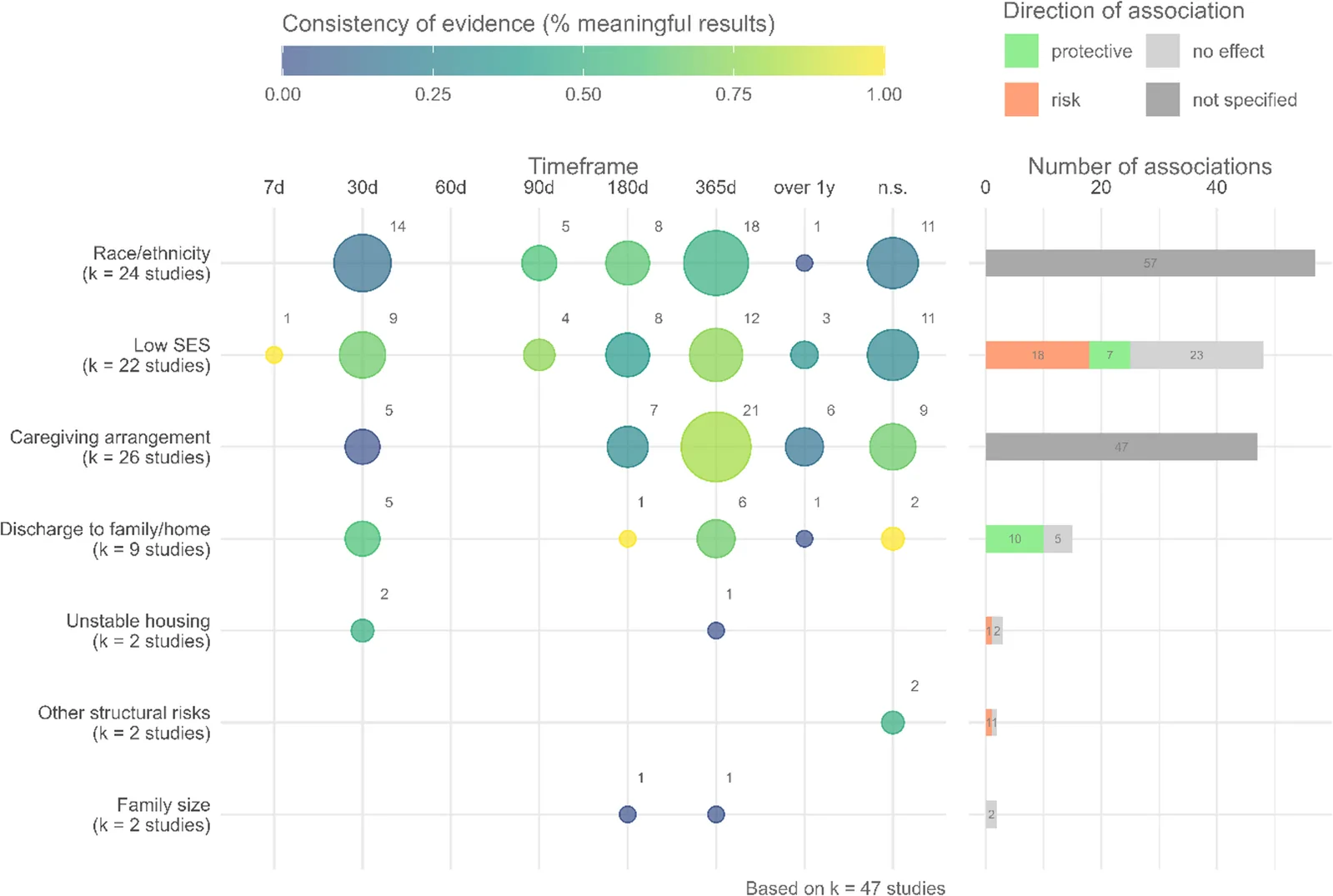
Quantitative findings on structural family variables. *Note.* One association within 28 days coded as 30-day timeframe for clear arrangement (ID 39). The size of the circles represents the number of associations, while the color reflects the relative strength of the evidence (see color legend above the figure). Direction of effect is not specified for race/ethnicity and caregiving arrangement due to different comparison groups. A detailed summary of subcategory associations can be found in the Online Resource S3

**Fig. 5:**
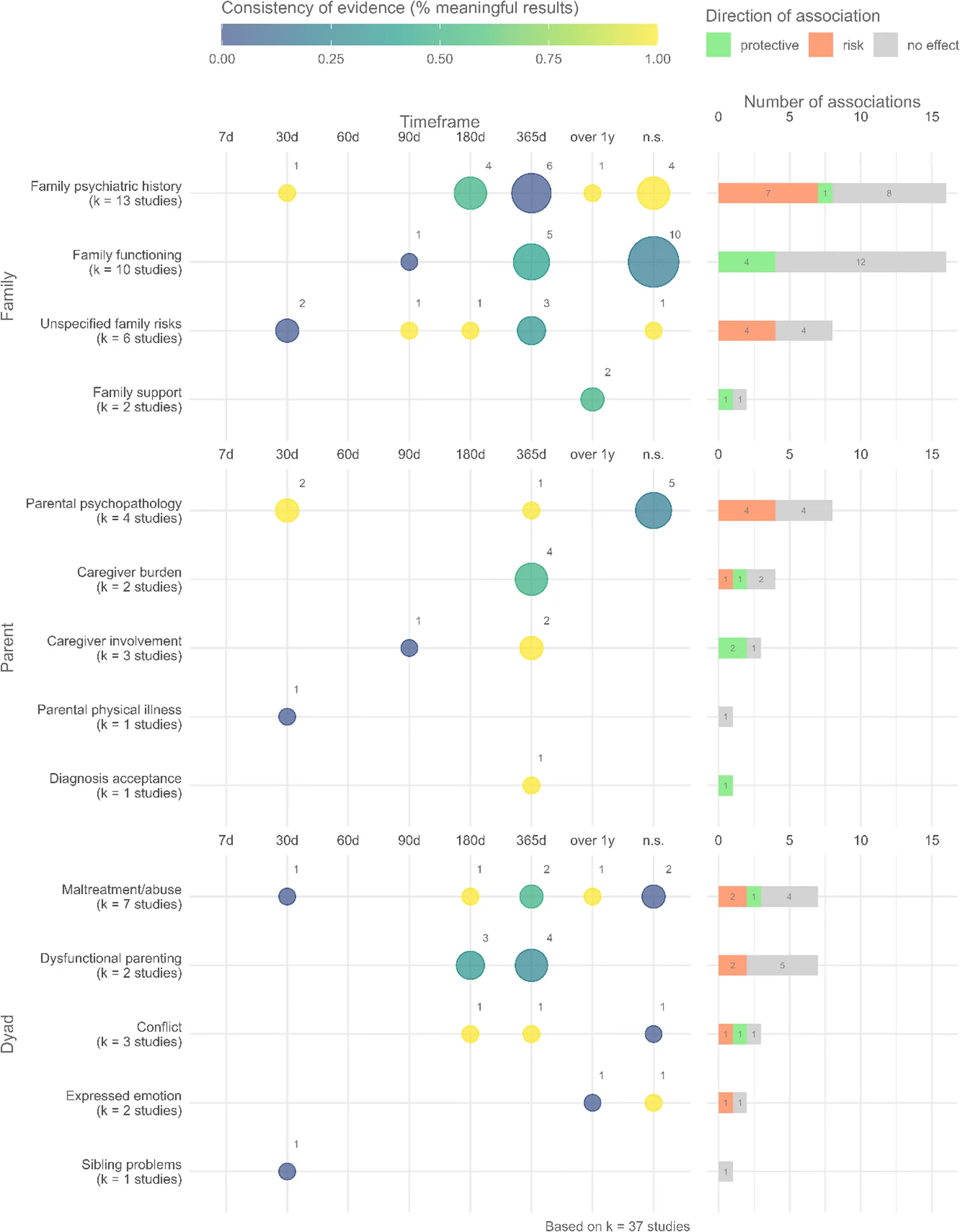
Quantitative findings on family process variables. *Note.* The size of the circles represents the number of associations, while the color reflects the relative strength of the evidence (see color legend above the figure)

**Fig. 6:**
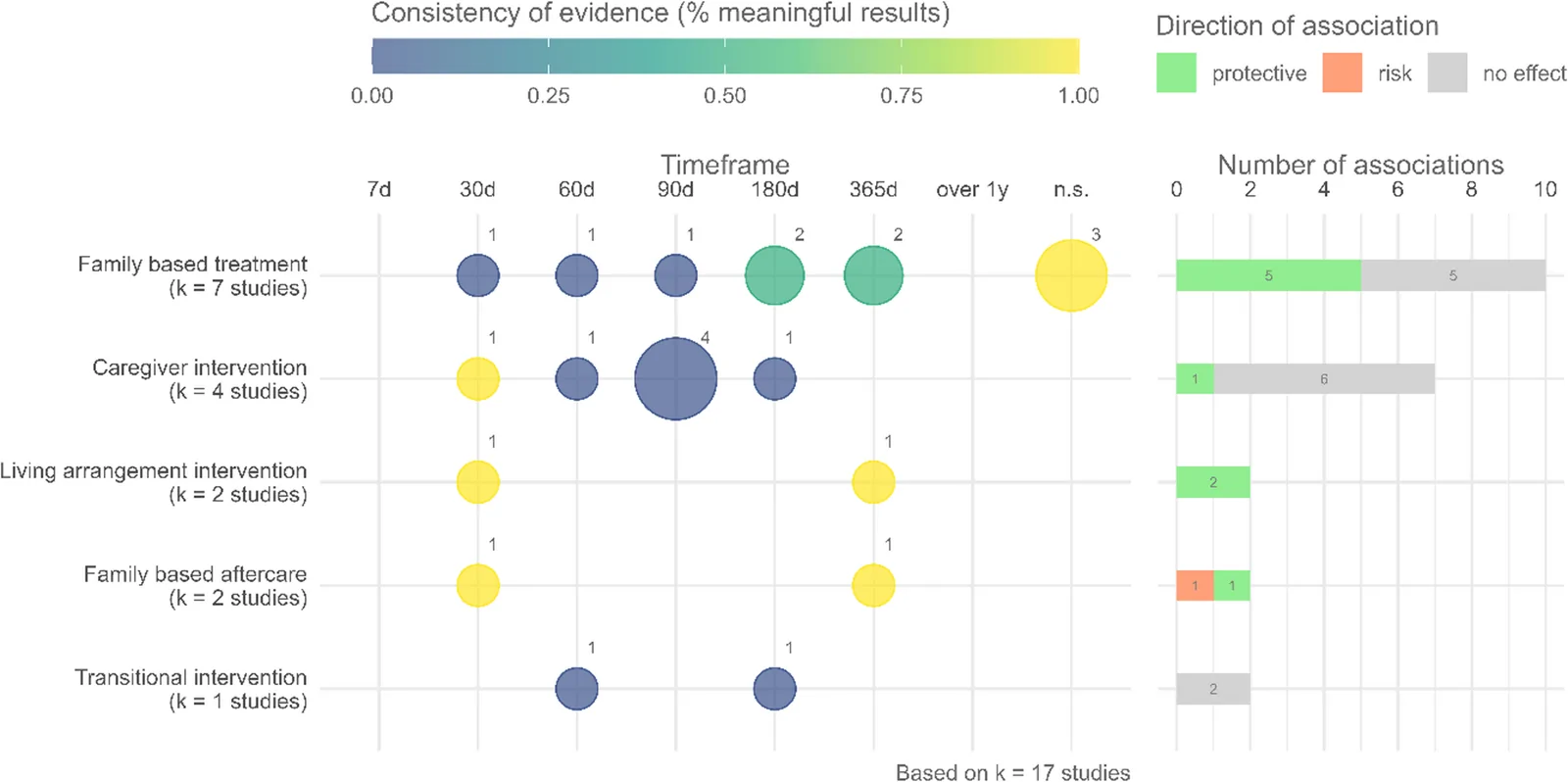
Quantitative findings on family-level interventions. *Note.* The size of the circles represents the number of associations, while the color reflects the relative strength of the evidence (see color legend above the figure)

Figures 4, 5 and 6 present the synthesized results of quantitative studies organized by the level of assessed family constructs (structural characteristics, process variables, family-based interventions). Three studies (IDs 48, 59, 74) were excluded from this summary, as they did not report any inferential tests or effect size estimates for the examined associations. Non-interventional results (254 individual associations from 59 studies) far exceeded interventional ones (23 individual results from 17 studies). Among non-interventional results, structural family characteristics were examined most frequently with 175 individual associations (47 studies) compared to 79 associations (37 studies) on the process level. Note that this synthesis often includes multiple associations from the same study. Constructs with high ratios of extracted associations to contributing studies included dysfunctional parenting (3.5 associations per study), race/ethnicity (2.4), socioeconomic status (2.2), caregiver burden (2.0) and parental psychopathology (2.0). All remaining constructs yielded fewer than two associations per study.

On the level of structural family characteristics (Fig. 4), examined constructs mostly pertained to race or ethnicity, indicators of socioeconomic status (SES), and caregiving arrangement. Family ethnic background as well as caregiving arrangements were operationalized differently across studies and could therefore not be summarized at an effect level. A detailed analysis of these subcategories can be found in Online Resource S3. There was a trend towards higher readmission risk for patients with minority backgrounds, although findings were overall mixed. Lower SES was associated with readmissions in just over half of cases (25/48). While about three quarters (18/25) of meaningful associations pointed to low SES as a risk factor for readmission, we also documented cases in which it served a protective function, suggesting further complexity in the relationship between SES indicators and readmission risk. Caregiving arrangements broadly characterized by the absence of one or both biological parents were largely not associated with readmissions (5/16). However, specific caregiving arrangements (e.g., adoption, foster or institutional care, single-parent families) often showed positive associations with readmissions.

Records of discharge disposition were examined in nine studies with regard to whether youth returned to family care or to another setting. Two-thirds of results (10/15) suggested that discharge to family was associated with a lower risk of readmission, whereas the remaining found no association. Six studies examined other characteristics of the caregiving context. One result each supported unstable housing and adolescent patients being single parents themselves as readmission risk factors. No meaningful associations were reported for family size.

Studies examined a range of constructs describing family processes (Fig. 5). We classified about half at the level of global family processes (54%), whereas dyad- (24%) and parent-level processes (22%) were examined considerably less. Family psychiatric history, family functioning, parental psychopathology, and maltreatment were the most examined constructs.

At the level of global family processes, studies found higher readmission rates for youth with a history of family psychiatric disorders in almost half (7/16) of reported results. Evidence was mixed for timeframes up to one year, whereas analyses without specified timeframes unanimously reported meaningful associations. Family functioning was meaningfully associated with lower readmission rates in a quarter of results (4/16), independent of the examined timeframe. Seven studies further examined nonspecific family risk measures based on clinical records or risk indices. Their findings suggest that some operationalizations of global family risk could predict subsequent readmissions (4/8). Of two studies examining family support, one showed a reduction in readmissions when family support was present.

Regarding parent-level family processes, having parents with documented psychopathology represented a meaningful readmission risk for youth in half of the findings (4/8). Caregiver burden was examined in several studies and yielded inconsistent results in terms of both meaningfulness as well as direction of effects. Treatment-related caregiver factors were highlighted in studies on caregiver involvement (2/3) and acceptance of a psychosomatic diagnosis (1/1). Parental physical illness was examined in one study and not associated with readmissions.

Dyadic processes involving the child, including parent-child and sibling dyads, were varied regarding their associations with readmission. Two studies examining parenting identified specific dysfunctional practices associated with a higher readmission risk, namely physical punishment and a permissive parenting style (2/7). Findings on parental maltreatment or abuse varied in relation to readmission risk (3/7) and direction of effect (2 risk, 1 protective). A similar pattern was apparent for parent-child conflict (2/3; 1 risk, 1 protective). Expressed emotion as a known risk factor in the adult literature was only examined in two studies, with one reporting meaningful and one reporting no association with readmission risk. One study examined patient-sibling relationships and reported no association of sibling problems with readmission risk.

#### Findings from quantitative studies: interventional results

Findings on family-based interventions (Fig. 6) included results from both controlled designs as well as naturalistic evaluations. Treatment programs explicitly focusing on and/or incorporating family intervention were effective at reducing readmission rates in half of examined associations (5/10), specifically over longer timeframes of six months and above. However, apart from one RCT (ID 71), these results were mostly based on naturalistic studies and specific to eating disorder patients, whereas controlled studies of broader patient populations often did not show reduced readmissions. Intervention elements that focused solely on caregivers largely did not impact readmission rates (1/7). Notably, these results were only based on timeframes up to 6 months. Reduced readmission rates were reported for interventions aimed at changing problematic living arrangements, both short-term within one month as well as up to one year after discharge (2/2).

Two studied aftercare programs directed at families were divergent in terms of readmission outcome (1 risk, 1 protective). Note that these results were based on non-controlled designs and included various forms of post-discharge care. An effective type of aftercare was case management for caregivers of youth identified to be at higher risk of readmission, which also included parenting support (ID 55). Results of studies examining programs that support families during the transition out of inpatient treatment did not provide evidence of their efficacy in reducing subsequent readmission risk.

#### Findings from qualitative studies

Studies using qualitative approaches mainly focused on the experiences with and reasons for readmissions. Prominent aspects of readmission experiences included dysfunctional relationships, neglect, lack of structure, aggression and violence, and lack of trust. Studies on patients’ perspectives reported their experience of a stressful family and home environment as a major post-discharge challenge and reason for return to inpatient care (IDs 19, 30, 32). Parental caregivers brought up unmet expectations regarding their children’s inpatient stay and return home as a primary reason for readmission, expressing a desire for more comprehensive care beyond acute stabilization (IDs 12, 32). Two studies reported that clinicians identified causes for readmission within the social and home environment more frequently than caregivers in the respective study (IDs 12, 28).

#### Findings from other sources

One meta-analytic systematic review (ID R1) on predictors of youth psychiatric readmission met our inclusion criteria (k = 33 studies, publication dates of primary studies covering 1997 to 2018). Meta-analytic summary effects of associations between examined family constructs (parental caregiver, family psychiatric history, abuse or neglect) and readmission risk were non-significant in all cases, but limited by a small number of included effect sizes (3 to 6).

The three study protocols included in the current review described evaluations of treatment approaches aimed at reducing readmissions through intensive family support (IDs P1, P2, P3; cf. Online Resource S2). While their respective data collection periods are officially concluded, results have not yet been published to our knowledge.

### RQ 2: Previous research practice and recommendations for the future

#### RQ 2a: Methods and sources of evidence

Online Resource S4 (cf. Figure S4.1) shows a summary of methods and sources of evidence that were utilized to gather family-related data in the included studies of this review. Regarding study methods, record data analysis was most prevalent (*k* = 50 studies, 63%). Other methods of similar frequency included interviews (*k* = 15 studies, 19%), interventions (*k* = 16 studies, 20%), and questionnaires (*k* = 13 studies, 16%), and one study using observation. Regarding the data source, administrative sources were drawn upon in two thirds of studies (*k* = 51 studies, 65%), while family constructs were less frequently reported by family members (*k* = 18 studies, 23%), clinicians (*k* = 17 studies, 22%), and patients (*k* = 12 studies, 15%). Six studies (8%) used researcher ratings. Regarding timeframes, non-interventional associations of family constructs with readmission outcomes were examined mostly over longer timeframes (i.e., six months and above, see Figs. 4 and 5). The timeframes for intervention outcomes were balanced more evenly up to one year (Fig. 6).

We further split studies into prospective and retrospective approaches. A total of 62 retrospective studies (78%) highlight the general dominance of this perspective. However, we attribute this predominance primarily to the high number of retrospective administrative record studies (see above), while other study approaches showed a more balanced distribution between retrospective and prospective designs. Visual inspection of the data showed a rise in retrospective studies over the past two decades, whereas the publication rate of prospective studies has remained relatively stable (see Figure S4.2 in Online Resource S4).

#### RQ 2b: Limitations and future recommendations

Online Resource S5 presents the frequency of reported themes and the corresponding IDs. Unexamined variables were the most common limitation that empirical studies reported (*k* = 34, 43%). Mentioned variables that could not be assessed mainly included (a) clinical and treatment characteristics (e.g., symptom severity and chronicity), service access and utilization, insurance status, and treatment adherence as well as (b) family and community factors, such as area socioeconomic conditions, caregiver burden, and parental mental health status or needs.

In addition, studies often reported sampling issues leading to potentially biased (*k* = 25, 32%) or underpowered (*k* = 23, 29%) samples. Several studies were further limited by their method of obtaining readmission data, often relying on institutional or regional records or recalled self-report. Thus, some readmissions may not have been captured in these studies, potentially leading to misestimation of overall readmission rates and of the effects of examined predictors. This limitation applies both to studies acknowledging the general possibility of uncaptured readmissions (*k* = 21, 27%) and specifically to single-site studies (*k* = 19, 24%). Similarly, the quality of available data on family variables was frequently reported as questionable, due to potentially unreliable (*k* = 18, 23%) or inaccurate (*k* = 15, 19%) sources and measures. This relates to potential inaccuracies of parent- or self-report, psychometric validity of the family measures used, as well as gaps and low level of detail in administrative data.

When facing issues around unavailable or inaccurate data, studies sometimes relied on proxy variables (*k* = 4, 5%). This primarily concerned indicators of socioeconomic status, which are inherently prone to bias, measurement error, and problems with construct validity. Design-related limitations that constrain causal inference were also noted. These included retrospective (*k* = 15, 19%), noncontrolled (*k* = 12, 15%), or non-randomized designs (*k* = 5, 6%) and broad, nonspecific interventions (*k* = 2, 3%).

The included meta-analytic review echoed these limitations, noting unexamined variables as inherent blind spots – particularly in relation to post-discharge care and social determinants of health, which were highlighted as underexplored gaps [1]. Moreover, as outlined in the introduction, most results on family-level constructs were incomparable across studies and could therefore not be meta-analytically synthesized.

To address these issues, recent publications have discussed recommendations to advance the field (see Table 5.2 in Online Resource S5). Themes were grouped into recommendations related to methodological advancements (methods), issues to examine further (examine), and developments in clinical practice (develop). Methodologically, study authors have acknowledged a need to expand the available data both in terms of scale (*k* = 9, 17%) and source (*k* = 5, 9%). This may be achieved by leveraging health registry or routine data sets as well as transregional and multi-center studies. Prospective (*k* = 6, 11%) and randomized and controlled (*k* = 2, 4%) designs have been highlighted as an opportunity to corroborate existing results. Advanced statistical methods potentially supporting these advancements (*k* = 3, 6%) that were mentioned include latent class analyses, growth modelling of trajectories over multiple timepoints, and machine learning approaches.

Research gaps identified by the included studies have further raised important questions for future research. First, an increased focus should be placed on post-discharge factors (*k* = 4, 7%), specifically community (*k* = 5, 9%) and caregiver (*k* = 5, 9%) factors, and their causal relation to readmission outcomes (*k* = 2, 4%). Second, specific risk factors, such as social disparities (*k* = 11, 20%), comorbidity (*k* = 4, 7%), and accessibility of healthcare services (*k* = 2, 4%) should be investigated further. Third, important treatment-related questions pertain to the effect of follow-up care (*k* = 12, 22%), family-focused interventions (*k* = 7, 13%), alternate treatment modalities (*k* = 4, 7%), and differential indication (*k* = 2, 4%). Last, it was pointed out that it is important to thoroughly examine the patients’ and their families’ experiences around hospitalization and surrounding care (*k* = 2, 4%) to better understand where improvement measures could be implemented. According to the authors of these studies, translating these research findings into clinical practice requires the adaptation of existing treatments (*k* = 8, 15%), the development of predictive tools and models (*k* = 6, 11%), and the implementation of personalized interventions (*k* = 2, 4%).

## Discussion

Reducing and preventing unplanned readmissions in child and adolescent psychiatric inpatient care is becoming increasingly relevant in light of a shift towards early reintegration into outpatient and family-based care. Readmissions thus represent cases in which improvements could not be maintained outside of intensive inpatient treatment. At the same time, readmission rates reflect mental health service outcomes that emerge from the interaction between individual need and healthcare system factors (e.g., healthcare policy, availability, and utilization). For youth, the family environment plays a pivotal role in this interaction by shaping subsequent recovery, access to follow-up care, and decisions around service use both directly and indirectly. Against this background, examining family factors seems crucial for understanding patterns and trajectories of repeated psychiatric hospitalizations. This study synthesized existing research on the role of different family factors and family-based interventions in youth psychiatric readmission, using a scoping review methodology to map the breadth of the field and inform future research.

Overall, a growing number of publications – particularly in recent years – demonstrate that research is paying increasing attention to the factors contributing to youth readmission at the family level. More specifically, the current findings suggest that family context variables can be understood as relevant but complex readmission factors, almost all showing some reported associations with varying degrees of consistency. At the same time, family constructs were operationalized very differently, limiting comparability. Structural characteristics of the caregiving environment were studied most extensively. Across various indicators, findings suggest increased readmission risk for youth returning to burdened or disrupted family contexts, including unstable, institutional or foster care environments. Results on family socioeconomic and ethnic minority status also showed associations with readmission risk, but without clear direction. This heterogeneity may partly arise from different study conceptualizations, populations, or designs, as well as the complex ways in which disparities interact with aspects of the healthcare system. Clarifying how and under what conditions these disparities affect readmissions will help us understand whether they indicate unequal access to post-discharge care (e.g., due to uneven regional distribution of healthcare and community resources or stigmatization and discrimination processes), or barriers to necessary but costly inpatient treatment (e.g., because of socioeconomic burden or limited insurance coverage).

Compared to structural characteristics of the family context, family process variables have been examined considerably less, and rarely in a methodologically adequate or comparable way. This gap reflects a reliance on variables readily captured in routine data, leaving more proximal family processes largely without adequate evidence despite their theoretical and practical relevance. As expected, some global family risk measures emerged as predictive for readmissions. Of note, this categorization spanned a broad range of constructs including a family history of psychiatric disorders, domestic violence, and general parenting problems. Family risk indices are yet to converge on common indicators and operationalizations. In contrast, indicators of high family functioning and support, operationalized by measures of general functioning, family cohesion, and relationship quality, reduced readmission risk in some studies. However, the predominance of null associations indicates that these measures did not reliably account for youth readmissions. Therefore, family functioning may act in conjunction with various other structural and process factors contributing to readmission risk and should be considered in clinical practice as part of a broader intervention strategy rather than as a sole, isolated target.

Regarding parent-child processes, studies found that permissive parenting practices and physical punishment were related to youth readmission. They reflect parental difficulties in managing their child’s behavior and are proven family risk factors for child development and negative symptom development [48, 49]. Highly distressed or abusive parent-child relationships also showed associations with readmission risk but yielded mixed results in terms of their direction. This finding contradicts previous knowledge that dysfunctional parent-child relationships belong to the strongest family risk factors for youth [21]. The parental gatekeeper role in determining youth service use may partly explain these inconclusive findings, in that problematic parent-child relationships may alter the utilization of services independent of clinical need. In line with our findings, a recent meta-analysis also showed inconsistent effects of youth history of abuse on readmission risk [1]. Furthermore, the examination of expressed emotion, although prominent in the adult literature, was almost completely absent from the studies we included. This may stem from the fact that EE conceptualizations have yet to be adequately translated and applied to research with youth populations. Indeed, the two included studies examining EE constructs used measures with unclear methodology and definition.

Family variables related to the parental level were examined the least. Consistent with previous literature identifying parental psychopathology as a general risk factor for their children’s mental health [50], some indicators of parental mental health problems showed association with youth readmission risk in our review. Moreover, several studies investigated parental stress and burden associated with caring for a hospitalized or mentally ill child or adolescent, with varied results. These inconsistencies may be attributed to different conceptualizations or methods in the dimensions of burden measured. Objective burden (i.e., observable demands of providing care) was a readmission risk in one study, likely reflecting the impact of strained resources. However, higher subjective burden (i.e., parental self-report of stress or worry) was related to reduced readmissions. This differential effect has been previously discussed, suggesting that parents’ internalized stress may reflect or motivate greater involvement in treatment [51]. Such parental involvement can greatly improve youth outcomes, and this review likewise showed reduced readmission risk when parents were involved and aligned with their children’s treatment. However, internalized guilt and shame can also hinder seeking help, such as a necessary rehospitalization [52]. Therefore, a differentiated assessment of parental burden and its associations with youth health outcomes warrants attention in both research and clinical practice.

Regarding interventions targeting the family context, research was largely observational, with little controlled data. Evidence of efficacy was documented for family-based treatments of eating disorders, which were associated with reduced longer-term readmission rates. This underscores that explicitly involving the family can improve the sustainability of treatment outcomes in children and adolescents. However, considerable heterogeneity exists even within eating disorder interventions, and little research was identified on patient populations with other or a broader range of conditions. The few other studies evaluated intensive family therapy embedded within complex crisis stabilization programs and did not demonstrate clear readmission effects. Such comprehensive approaches better reflect clinical reality, but their multi-component nature precludes attribution of outcome effects to any specific element. Nevertheless, these studies demonstrate that innovative treatment programs incorporate a family focus and that the field seems to move toward greater methodological rigor.

Interventions specifically targeting parents included various forms of support, such as separate sessions, parent training, or peer groups that also addressed other outcomes beyond readmissions. In our review, these parent-focused interventions were largely ineffective at preventing readmissions. Nevertheless, some studies report meaningful effects and nominal decreases, suggesting potential for improvement on this level.

Qualitative studies complement the quantitative research that is more prevalent in this field. They revealed practically relevant discrepancies between caregiver and clinician perspectives on repeated hospitalizations, as each side placed readmission-related issues outside their area of responsibility. To close this gap, the collaboration between the clinic and home must be promoted by actively engaging caregivers in treatment, setting realistic expectations, and providing close support during the hospital-to-home transition to ensure continuous, adequate post-discharge care.

### Limitations of the primary studies

Several characteristics of the included studies shape the conclusions of this review. First, the growth of literature in this area in recent years was mostly driven by retrospective studies of incidental (e.g., administrative record) data, whereas the number of prospective studies has remained modest and relatively stable over time. This leaves important aspects of the social context of children and adolescents relatively understudied [31], as is evident in the predominance of studies focusing on structural aspects of the caregiving environment (commonly captured in routine datasets), while in-depth investigations into family processes and interactions received less attention.

Second, most studies were conducted in the United States, Australia, and Canada, while research from Europe, Asia, Africa, or Latin America was scarce. This imbalance represents a significant gap in the understanding of youth readmissions as an outcome within the context of global healthcare systems and sociocultural conditions. Moreover, studies of family factors primarily investigated timeframes of six months and above. While these longer-term studies provide valuable data on sustained outpatient stability, the relative lack of short-term studies contrasts with the fact that readmissions most often occur within the first three months post-discharge. As a result, this gap substantially limits the extent to which current evidence can inform conclusions about which family-related factors are most relevant during this critical period.

Third, readmission outcomes of interventions targeting the family environment were mostly evaluated naturalistically, with only a few controlled trials. This means that in some cases, associations may be confounded with clinical need or available resources rather than indicating a direct intervention effect. For example, conflicting results on the receipt of aftercare may be explained by heightened need, general access to services, or intervention heterogeneity. Similarly, reduced readmissions following changes in the youth’s living situation suggest benefits of intervention in the case of environmental risks but have not been compared against equally severe cases without this intervention.

### Implications for research and practice

The complex picture revealed in this review underscores the potential of expanding research on family-related factors to improve post-discharge care and reduce inpatient readmissions in child and adolescent psychiatry. Yet, as apparent from the study findings, family-level factors are still rarely part of routine datasets and research. The inclusion of standardized assessments of psychosocial information in routine data would be necessary to close this gap. Previous reviews similarly highlight the relative scarcity of research on community- and family-level variables and the need for standardized measures [1, 23, 24]. In addition, prospective studies are needed to capture outcome trajectories and long-term family predictors in detail.

This review highlights that post-discharge factors at the parental and parent-child level are particularly understudied compared to their prominence in the broader clinical family literature. Results partly align with known risk factors for youth mental health, such as family psychiatric history and parental psychopathology [50, 53], dysfunctional parent-child and family relationships [20], and generally stressful family contexts [21]. For readmissions as a marker of recurrent relapse, findings from the present review only partly supported their relevance for post-discharge outcomes. However, we believe that research will greatly benefit from increasingly applying the well-established family psychology and parent-child literature to this specific research area. This includes more intensive research on specific parenting behaviors and parent-child interactions, parental stress and caregiver burden, as well as the application of established parenting trainings and interventions (e.g. [54]), .

Overall, the family environment is a key lever in the treatment of children and adolescents to achieve sustained improvements, as family processes are transdiagnostically relevant and have been proven to be modifiable through intervention [15, 24, 55]. Current evidence suggests that clinicians aiming to reduce readmission risk may consider engaging families early during discharge planning, assessing structural burdens and support needs, and collaborating with caregivers to strengthen cohesion and alignment with treatment goals. Establishing evidence-based family-focused care in treatment protocols, ideally through randomized controlled trials, will allow for testing the efficacy of various components in improving youth relapse, readmission, and recovery rates. This applies to inpatient, transitional, and post-discharge care as well as approaches that explicitly avoid hospitalization, such as outpatient day clinics and home treatment. Protocols for three controlled intervention studies identified in this review (IDs P1- P3) suggest that these necessary developments are progressing.

### Limitations of this review

We outlined the rationale for our approach and methodological decisions taken herein but acknowledge that they come with some limitations that are important to consider. First, the selection of records was limited to English and German literature. Research documented in other languages might thus have been missed, contributing to the overrepresentation of studies in English-speaking countries within our sample.

Second, we extracted multiple associations between family-related variables and readmission for studies reporting more than one, for example for multiple comparison categories or separate analyses per timeframe. We opted for this approach to gather a complete overview of research findings. However, this may overemphasize studies reporting on multiple associations for the same family-related construct. Of note, this was mostly the case for multiple comparison categories (i.e., ethnicities, income quantiles, caregiving arrangements), where only a few showed meaningful associations. Therefore, our count of meaningful associations represents a conservative lower limit as compared to a strategy where only one result (i.e., a meaningful result) would have been assessed.

Third, our definition of meaningful associations meant that some nonsignificant findings were classified as meaningful if their effect sizes exceeded our chosen threshold for a non-trivial association (*r* > .10) and significant findings could be trivial in effect strength in overpowered studies. Critically, our results should not be interpreted as a substitute for a rigorous meta-analytic estimation of a true population effect (or an investigation into effect moderators). Our goal was to provide readers with a useful signal-to-noise ratio in focusing on potentially meaningful candidate variables in future works. To this end, our approach represents the practical relevance of individual results to a more comprehensive degree than statistical significance alone, especially given the power issues in many of the included studies.

Last, including both bivariate and multivariate results introduced variation in cross-study comparisons. To maximize consistency, we extracted bivariate associations where possible but also included some studies that only reported multivariate results. Associations were synthesized across both, as no clear differences emerged except for structural variables contributing to readmission slightly more often in multivariate analyses. This may stem from higher-powered studies including multiple variables while simultaneously increasing the rate of significant results. Conversely, simple bivariate analyses may underestimate the complexity of structural characteristics’ influences on youth readmission risk. Future meta-analytic work could thus investigate moderator variables, such as service availability, symptom severity, or health literacy. A detailed comparison of bivariate and multivariate associations can be found in Online Resource S3.

## Conclusion

This review illustrates the growing research interest in family factors after youth psychiatric inpatient treatment, but at the same time identified substantial gaps in prospective studies outside of the US healthcare context and in-depth examinations of family processes. Overall, findings suggest that readmission risk is altered for youth returning to structurally burdened families and families displaying impaired interpersonal processes. To better understand psychiatric readmissions and the role of the family context in subsequent utilization patterns, research needs to clarify social disparities, expand upon post-discharge and caregiver factors, and evaluate family-oriented interventions aimed at translating scientific findings into clinical practice. These advancements require dedicated prospective designs that are well informed by the wealth of the relevant family psychology literature.

## Supplementary Information

Below is the link to the electronic supplementary material.

Online Resource S1 (PDF 163 KB)

Online Resource S2 (PDF 318 KB)

Online Resource S3 (PDF 636 KB)

Online Resource S4 (PDF 316 KB)

Online Resource S5 (PDF 118 KB)

## Data Availability

No datasets were generated or analysed during the current study.
