# Supplementary material for: The role of the family in youth psychiatric readmission: a scoping review
Source: Eur Child Adolesc Psychiatry. 2026 Jun 6;35(8):2349–65. doi: 10.1007/s00787-026-03062-y (PMC13652881; doi:10.1007/s00787-026-03062-y)
Supplement: Supplementary file 1 [file 787_2026_3062_MOESM1_ESM.pdf]

Online Resource 2

Characteristics and reference list of included studies

Overview

Table S2. Characteristics of included studies (k = 83) grouped by study type

References. APA-style bibliographic references to included studies

Corresponding author: Amos-Silvio E. Friedrich, University of Vienna (amos.friedrich@univie.ac.at)

Table S2 Characteristics of included studies (k = 83) grouped by study type

ID	Authors (Year)	Country	Data collection period	Sample	Readmission rate[a]	Study design	Family constructs/interventions examined
**Empirical Studies**							
1	Arnold et al. (2003)	USA	1991 – 2002	*N* = 180 adolescents (12 to 19 years) with no intellectual disability or systemic physical diagnosis admitted for min. 10 days	6m: 19%; 24m: 33%; 120m: 49%	Quantitative	Race/ethnicity
2	Barker et al. (2010)	Australia	2005	*N* = 112 young people (11 to 17 years) with severe MH issues	31%	Quantitative	Race/ethnicity; family psychiatric history; discharge disposition; history of abuse or neglect
3	Behere et al. (2017)	USA	2012	*N* = 143 children (≤12 years) admitted for 24 hours	n.r.	Quantitative	Race/ethnicity; family structure, family psychiatric history
4	Blader (2004)	USA	n.r.	*N* = 109 children (5 to 12 years) admitted during a 14-month period	34%	Quantitative	Race/ethnicity; caregiver (biological, adoptive, grand, foster parents); parental involvement; corporal punishment; poor monitoring; rewards positive behavior; inconsistent discipline; parenting stress; family control; family cohesion
5	Bloom & Hopewell (1982)	USA	1976 – 1979	*N* = 88 adolescents discharged from a state mental hospital	43%	Quantitative	Race/ethnicity; hospitalization of a family member; family structure, number of siblings; discharge living arrangement
6	Bobier & Warwick (2005)	New Zealand	2001 – 2003	*N* = 71 patients admitted to a youth inpatient unit	48%	Quantitative	Unstable accommodation; covert family dynamics; parent-child conflict
7	Brown et al. (2023)	South Africa	2013 – 2017	*N* = 189 adolescent patients (13 to 18 years) admitted to a psychiatric hospital	18%	Quantitative	Family upbringing problems; family history of psychiatric disorders; primary caregiver
8	Carroll et al. (2022)	USA	2016 – 2018	*N* = 23,556 children (5 to 18 years) with mental health conditions	6%	Quantitative	Race/ethnicity; private/government insurance; household income

ID	Authors (Year)	Country	Data collection period	Sample	Readmission rate[a]	Study design	Family constructs/interventions examined
9	Charlemagne (2012)	USA	n.r.	*N* = 167 children and adolescents (≤ 18 years) admitted for 24 hours or more at a psychiatric hospital	26%	Quantitative	Race/ethnicity; family involvement
10	Chung et al. (2008)	USA	2004	*N* = 403 children and adolescents admitted to an inpatient psychiatric hospital	16%	Quantitative	Race/ethnicity; living arrangement
11	Connell et al. (2021)	USA	2014 – 2016	*N* = 1,090 children and adolescents (5 to 17 years) admitted for an inpatient stay with SI or self-harm	All-cause: 8%; Same-cause: 2%	Quantitative	Caregiver counseling on lethal means restriction
12	Connell et al. (2021)	USA	2017 – 2018	Parents and/or legal guardians, attending psychiatrists of readmitted patients with MH conditions	n.a.	Qualitative	Discordant expectations; discharge hesitancy; challenging social environment; challenging home environment
13	Cotton (2025)	USA	2024	*N* = 42 adolescents discharged from a behavioral health unit	Before: 22%; After: 0%	Quantitative	IDEAL transitional intervention including caregivers in discharge planning
14	Czyz et al. (2016)	USA	2002 – 2006	*N* = 373 adolescents (13 to 17 years) psychiatrically admitted because of acute SI or a recent suicide attempt	21%	Quantitative	Family connectedness
15	D'Aiello et al. (2021)	Italy	2017 – 2019	*N* = 224 children and adolescents hospitalized for the first time over the 3 selected years	23%	Quantitative	Family history of psychiatric disorders; adoption; discharge disposition
16	Day et al. (2025)	USA	2011 – 2016	*N* = 1,370 youth (5 to 12 years) admitted to a child psychiatry unit	8%	Quantitative	Race/ethnicity; SES (insurance); guardianship; stable/unstable housing
17	Deolmi et al. (2021)	Italy	2013 – 2015	*N* = 51 adolescents (12 to 17 years)	n.r.	Quantitative	Protective/unprotective family environment; living arrangement
18	Eichstadt et al. (2023)	South Africa	2015 – 2019	*N* = 345 adolescent inpatients (11 to 18 years)	28%	Quantitative	Parental mental illness; parent-child conflict

ID	Authors (Year)	Country	Data collection period	Sample	Readmission rate[a]	Study design	Family constructs/interventions examined
19	Ekwemalor et al. (2017)	USA	n.r.	*N* = 16 readmitted adolescents (13 to 17 years) with at least one mental illness	n.a.	Qualitative	Problematic parental relations; high-risk family; living environments
20	Feng et al. (2017)	USA	2014	*N* = 47,397 (5 to 17 years) discharged from community hospitals	8%	Quantitative	Race/ethnicity; SES (insurance)
21	Fite et al. (2009)	USA	n.r.	*N* = 80 children (6 to 12 years) with previous psychiatric inpatient treatment	19%	Quantitative	Parenting style
22	Fontanella (2008)	USA	1997 – 1998	*N* = 522 adolescents admitted to three private psychiatric hospitals	38%	Quantitative	Race/ethnicity; family risk index (parental history of mental illness, parental history of alcohol or drug abuse, history of family violence, severe family dysfunction); discharge disposition
23	Fontanella et al. (2010)	USA	1997 – 1998	*N* = 517 Medicaid-covered adolescents consecutively admitted to private psychiatric hospitals	12%	Quantitative	Race/ethnicity; discharge disposition; change in living arrangement
24	Foster (1999)	USA	1991 – 1993	*N* = 204 children and adolescents discharged from inpatient facilities during study period	n.r.	Quantitative	Race/ethnicity; SES (family income); SES (caretaker education), family structure; caregiver previously hospitalized; objective burden of care; subjective burden of care
25	Franklin et al. (2025)	USA	2021 – 2023	*N* = 46 adolescents (10 to 17 years) with anorexia nervosa admitted to an eating disorder unit receiving or not receiving FN (n_{IG} = 23, n_{CG} = 23)	7%	Quantitative	Family Navigator (FN) intervention offering transitional parent support
26	Gao et al. (2018)	Australia	2013 – 2014	*N* = 53 adolescents with somatic disorders	25%	Quantitative	Parental acceptance of somatic disorders diagnosis

ID	Authors (Year)	Country	Data collection period	Sample	Readmission rate[a]	Study design	Family constructs/interventions examined
27	Gearing et al. (2009)	Canada	1999 – 2003	*N* = 87 children and adolescents (< 18 years) hospitalized for their first episode of a primary psychotic disorder or a mood disorder with psychotic features	57%	Quantitative	Family history of psychiatric disorders; pattern of family criticism
28	Grudnikoff et al. (2019)	USA	2014 – 2015	*N* = 1,233 children and adolescents (5 to 17.9 years) admitted to psychiatric inpatient facility	30d: 11%; 365d: 18%	Mixed methods	Race/ethnicity; SES (income); SES (insurance); discharge to home/residential/group home
29	Herrmann et al. (2025)	United Kingdom	2018 – 2021	*N* = 184 adolescents (10 to 18 years) undergoing inpatient treatment for AN	8%	Quantitative	Newsbridge discharge prognosis score (NPS) including family burden and parental insight and support
30	Hillebrand (1991)	USA	1990	*N* = 10 female adolescents (12 to 16 years) rehospitalized for emotional problems	100%	Qualitative	Parent-child relationship; family visits; family history
31	Hughes et al. (2014)	Australia	2006 – 2010	*N* = 430 adolescents admitted for AN to a tertiary pediatric facility	Before: 47%; FBT: 37%	Quantitative	Family-based treatment
32	January et al. (2018)	USA	n.r.	*N* = 7 youth (9 to 18 years) readmitted to a therapeutic residential care program	n.a.	Qualitative	Family relationships
33	Kagabo (2016)	USA	1999 – 2010	*N* = 7,265 children and adolescents (3 to 17 years) at a pediatric psychiatric hospital	22%	Quantitative	Race/ethnicity; insurance type; discharge disposition
34	King et al. (1997)	USA	n.r.	*N* = 89 adolescents (*M* = 15.3 years) in psychiatric hospitalization	n.r.	Quantitative	SES (education, employment); caregiving structure; parent-child conflict; family functioning
35	Lakin et al. (2004)	USA	1998 – 2000	*N* = 89 children and adolescents (5 to 17 years) with severe behavioral problems receiving residential treatment	34%	Quantitative	Parental involvement; family functioning

ID	Authors (Year)	Country	Data collection period	Sample	Readmission rate[a]	Study design	Family constructs/interventions examined
36	Lammers et al. (2019)	USA	2010 – 2015	*N* = 2,492 medicaid-enrolled children admitted to inpatient care	IG: 10.2%; CG: 11.2%	Quantitative	Parent-partner intervention consisting of trained peer-to-peer support and limited posthospitalization care management
37	Lapointe et al. (2010)	USA	2007 – 2008	*N* = 664 low-income, inner-city ethnic minority youth (8 to 18 years) admitted to psychiatric care	13%	Quantitative	Race/ethnicity; caregiver status
38	Lavanier (2009)	USA	2006	*N* = 255 children (3 to 12 years) who were admitted to a public psychiatric inpatient unit	Study 1: 32%; Study 2: 39%	Quantitative	Race/ethnicity; insurance type; family functioning; abuse; family crowding; caregiver instability; parental psychopathology
39	Li et al. (2021)	Australia	2017	*N* = 200 children and adolescents (9 to 18 years) with AN or atypical AN as a primary or secondary diagnosis who were admitted to a public hospital	8%	Quantitative	SES (area socioeconomic status)
40	Lohr et al. (2024)	USA	2019	*N* = 66,710 (patients (5 to 17 years) in a state inpatient database with a primary MH diagnosis	8%	Quantitative	insurance type; SES (household income)
41	Lui et al. (2024)	USA	2017 – 2020	*N* = 6,908 youth (≤ 18 years) who received mobile crisis response services	15%	Quantitative	Race/ethnicity; insurance type
42	Markota et al. (2018)	USA	2015	*N* = 366 children and adolescents (11 to 18 years) without admission in the previous year (excluding eating, schizophrenia, bipolar, schizoaffective or psychotic disorders)	20%	Quantitative	Disrupted family environment
43	Mendes et al. (2017)	Portugal	2010 – 2013	*N* = 51 readmitted and *N* = 58 non-readmitted youth (≤ 18 years)	30d: 1%; 12m: 12%	Quantitative	Family history of mental illness; parental primary caregiver

ID	Authors (Year)	Country	Data collection period	Sample	Readmission rate[a]	Study design	Family constructs/interventions examined
44	Milliren et al. (2024)	USA	2017 – 2022	*N* = 1,288 patients (≥ 3) with avoidant-restrictive food intake disorder discharged from tertiary care hospitals in a clinical database	9%	Quantitative	Race/ethnicity; SES (insurance)
45	Miller et al. (2020)	Canada	2003 – 2014	*N* = 3,825 children and adolescents (3 to 19 years) admitted for psychiatric conditions	28%	Quantitative	SES (neighborhood income); family support (referral source, discharge to home)
46	Minchew (2006)	USA	2001 – 2003	*N* = 150 male adolescents (13 to 17 years) admitted to an acute inpatient psychiatric unit with at least one previous psychiatric hospitalization	n.a.	Quantitative	Family psychiatric history; discharge disposition; absence of biological parents; family conflict; childhood abuse
47	Mumbere et al. (2022)	Dem. Rep. Congo	2020 – 2021	*N* = 36 adolescent patients (10 to 17 years) free of active symptoms of severe mental illness, with a history of being discharged, and then readmitted at least one time for mental illness	n.a.	Quantitative	Expressed Emotion
48	Ney & Mills (1976)	Canada	1972 – 1976	*N* = 350 children (< 15 years) with emotional disturbances or behavior problems	6%	Quantitative	Family-focused treatment program
49	Oltean et al. (2020)	Canada	2015 – 2017	*N* = 100 children and adolescents (8 to 17 years) receiving MH treatment from a tertiary care facility and their parents	34%	Quantitative	Family functioning
50	Otterson et al. (2021)	USA	2017	*N* = 118 (12 to 18 years) admitted for SI or suicide attempt	14%	Quantitative	Crisis stabilization unit focusing on intensive treatment involving the family
51	Pavkov et al. (1997)	USA	1987 – 1992	*N* = 3,969 children and adolescents (7 to 17 years) admitted for the first time to inpatient psychiatric care	n.r.	Quantitative	Race/ethnicity; SES (family median income); SES (area socioeconomic index)

ID	Authors (Year)	Country	Data collection period	Sample	Readmission rate[a]	Study design	Family constructs/interventions examined
52	Persi & Sisson (2008)	Canada	1999 – 2004	*N* = 76 children in foster care and *N* = 540 children admitted to inpatient psychiatric care	Foster care: 40%; Comparison: 17%	Quantitative	Foster care
53	Phillips et al. (2020)	USA	2009 – 2010	*N* = 6,797 youth (6 to 17 years) admitted to psychiatric hospitals with a primary diagnosis of mood disorder	21%	Quantitative	Race/ethnicity; medicaid eligibility
54	Pieterse et al. (2016)	South Africa	2010 – 2011	*N* = 97 discharged adolescents (12 to 19 years)	36%	Quantitative	Living arrangement
55	Ramsbottom & Farmer (2018)	USA	2014 – 2017	*N* = 1,221 children (2 to 12 years) with risk of readmission	6%	Quantitative	Family mental health history; SES (insurance)
56	Rhodes & Madden (2005)	Australia	2001 – 2004	*N* = 144 youth hospitalized for AN receiving treatment before (*n* = 66) or after (*n* = 78) the introduction a FBT	Before: 26%; FBT: 9%	Quantitative	Family-based treatment
57	Sams et al. (2023)	USA	2019 – 2021	*N* = 119 adolescents (12 to 17 years) with no prior psychiatric hospitalization receiving rapid stabilization (*n* = 30) or treatment as usual (*n* = 89)	RSP 30d/90d/180d: 3%/17%/20%<br><br>CG 30d/90d/180d: 5%/14%/16%	Quantitative	Rapid Stabilization Pathway (RSP) intervention including intensive family therapy
58	Seleem & Amer (2021)	Egypt	2017 – 2019	*N* = 1,195 children and adolescents admitted to inpatient or outpatient psychiatric care	27%	Quantitative	Family status; parental education; family income; physical/sexual abuse
59	Sheifer et al. (2024)	Russia	2023	*N* = 76 adolescents (13 to 17 years) hospitalized due to suicidal ideation or attempt	45%	Quantitative	Family type; family psychiatric history; family functioning
60	Similä et al. (2018)	Finland	2001 – 2006	*N* = 403 adolescents (< 18 years) in need of acute psychiatric inpatient care	41%	Quantitative	Family structure; SES (caregiver education); SES (caregiver employment); caregiver psychiatric problems; caregiver substance abuse problems

ID	Authors (Year)	Country	Data collection period	Sample	Readmission rate[a]	Study design	Family constructs/interventions examined
61	Simmons et al. (2022)	USA	2019 – 2022	*N* = 238 discharged youth (< 17 years)	26%	Quantitative	Family-centered discharge intervention (CARE Community Advocacy and Resource Engagement)
62	Solerdelcoll et al. (2024)	Spain	2014 – 2017	*N* = 89 youth in foster care and *N* = 247 youth living with their immediate family hospitalized	25%	Quantitative	Foster care
63	Soutullo et al. (2023)	USA	2017 – 2019	*N* = 1,604 pediatric patients (4 to 17 years) with no admission within a year before index admission	15%	Quantitative	Race/ethnicity; SES (insurance)
64	Steinhausen et al. (2008)	Germany, Switzerland, Bulgaria, Romania	1979 – 1996	*N* = 212 patients (10 to 18 years) with AN	45%	Quantitative	Paternal alcoholism; history of AN in the family
65	Stewart et al. (2014)	Canada	2005 – 2010	*N* = 2,445 youth (12 to 19 years) admitted into adult psychiatric beds	43%	Quantitative	SES (race/ethnicity); living in group home or assisted care; dysfunctional family
66	Stoner (2014)	USA	2009 – 2010	*N* = 226 youth (4 to 12 years) admitted to a specialized private psychiatric inpatient unit	26%	Quantitative	Family functioning
67	Vadén et al. (2024)	Finland	2016 – 2020	*N* = 1,427 patients (13 to 17 years) admitted to an adolescent psychiatric ward	30d: 13%; To adulthood: 48%	Quantitative	Family risk factors (family violence, parental substance use problems, parental divorce or separation, bereavement, parental severe somatic illness, parental severe mental disorder, severe financial difficulties, severe problems related to siblings, [suspected] sexual abuse within family and other)
68	Van Alphen et al. (2017)	USA	2013 – 2014	*N* = 165 adolescents (13 to 19 years) with current major depressive disorder or dysthymia admitted to short-term residential treatment	12%	Quantitative	Race/ethnicity; SES (parental education); adoption; childhood maltreatment/abuse

ID	Authors (Year)	Country	Data collection period	Sample	Readmission rate[a]	Study design	Family constructs/interventions examined
69	Wallin & Holmer (2021)	Sweden	1990 – 2009	*N* = 68 youth (9 to 17 years) admitted patients with AN diagnosis	FBT 6m/12m: 5%/21% CG 6m/12m: 32%/32%	Quantitative	Family-based treatment
70	Wallis et al. (2007)	Australia	2001 – 2006	Individuals with AN (< 19 years) with a duration of illness of less than three years	n.r.	Quantitative	Family therapy
71	Wallis et al. (2018)	Australia	2007 – 2010	*N* = 69 medically unstable adolescents with AN	n.r.	Quantitative	Family-based treatment
72	Wang et al. (2024)	China	2022 – 2023	*N* = 235 adolescent inpatients (11 to 18 years) with affective disorders	n.r.	Quantitative	Family functioning
73	Ward et al. (2025)	United Kingdom	2012 – 2022	*N* = 239,541 children and youth (5 to 18 years) admitted to acute medical ward for mental health reasons	13%	Quantitative	Race/ethnicity; SES (regional index of multiple deprivation)
74	Winner et al. (2025)	USA	2020 – 2023	Pediatric behavioral health unit patients admitted due to suicidal or homicidal attempts, active mania or psychosis	Before 7d: 5%; 30d :12%	Quantitative	Interpersonal conflict; SES (insurance); conflict management skills; family therapy
75	Yampolskaya et al. (2014)	USA	2004 – 2008	*N* = 1,432 youth (*M* = 13.83 years) admitted to a state inpatient psychiatric program (SIPP)	1y: 10%; 4y: 19%	Quantitative	Race/ethnicity; involvement with child protection system; child maltreatment (type, severity, chronicity); absence of a caregiver; child welfare out-of-home-placement; parental substance abuse problems
76	Yampolskaya et al. (2013)	USA	2004 – 2008	*N* = 1,432 youth (*M* = 13.83 years) admitted to a state inpatient psychiatric program (SIPP)	Subsequent: 5%; 1y: 10%; Total: 19%	Quantitative	Maltreatment history
77	Yen et al. (2014)	USA	2006 – 2010	*N* = 99 adolescent (*M* = 15.3 years) psychiatric inpatients hospitalized due to a risk of suicide (suicidal behavior or severe SI)	28%	Quantitative	Family history of mood disorders

ID	Authors (Year)	Country	Data collection period	Sample	Readmission rate[a]	Study design	Family constructs/interventions examined
78	Yen et al. (2019)	USA	2012 – 2014	*N* = 50 adolescents (12 to 18 years) admitted to the inpatient psychiatric unit on the basis of suicide risk (attempt or SI)	32%	Quantitative	CLASP adaptation (parental involvement)
79	Yu et al. (2022)	USA	2012 – 2014	*N* = 36,857 children (≤ 18 years) with a primary mental health diagnosis discharged from hospitals included in PHIS database	9%	Quantitative	SES (neighbourhood opportunity index)
**Study Protocols**							
**P1**	Neumann et al. (2021)	Germany	2018 – 2021	Patients treated at a model hospital implementing FIT (IG) and patients treated at structurally comparable hospitals (CG)	Evaluate effectiveness of a flexible integrated treatment model project[b]	Mixed methods	Early-discharge intensive outpatient treatment focusing on family and caregiver support
**P2**	Falcone (2017)	USA	2017 – 2021	Patients (10 to 18 years) admitted to an inpatient psychiatry unit for suicidal ideation, behaviour, or attempt	Determine what combination of services is best suited to reduce subsequent suicide attempts and rehospitalization	Quantitative (RCT)	Wraparound service involving social network

ID	Authors (Year)	Country	Data collection period	Sample	Readmission rate[a]	Study design	Family constructs/interventions examined
P3	Dahmen et al. (2024)	Germany	2021 – 2025	Patients (12 to 18 years) diagnosed and admitted to inpatient treatment for anorexia nervosa	Test the efficacy and safety of early multidisciplinary home treatment involving caregivers	Quantitative (RCT)	Hometreatment involving caregivers
**Systematic Reviews**							
R**1**	Edgcomb et al. (2020)	-	1997 – 2018	33 studies reporting on quantitative predictors of psychiatric readmission for a sample of subsample of children and adolescents (< 18 years)	Investigate predictors of psychiatric readmission among children and adolescents	Quantitative (Meta-analysis)	Primary caregiver; family psychiatric history; history of abuse/neglect; discharge disposition

Note. AN = Anorexia Nervosa; CG = Control group; d = days; FBT = Family-based treatment; IG = Intervention group; m = months; MH = Mental health; n.a. = not applicable; n.r. = not reported; SI = Suicidal ideation; y = years.

[a] Rounded to the nearest whole number.

[b] Summary by the review authors (otherwise, aims are author-reported).

References

Arnold, E. M., Goldston, D. B., Ruggiero, A., Reboussin, B. A., Daniel, S. S., & Hickman, E. A. (2003). Rates and predictors of rehospitalization among formerly hospitalized adolescents. *Psychiatric Services*, *54*(7), 994–998. https://doi.org/10.1176/appi.ps.54.7.994

Barker, D., Jairam, R., Rocca, A., Goddard, L., & Matthey, S. (2010). Why do adolescents return to an acute psychiatric unit? *Australasian Psychiatry*, *18*(6), 551–555. https://doi.org/10.3109/10398562.2010.501380

Behere, A. P., Basnet, P., & Campbell, P. (2017). Effects of family structure on mental health of children: A preliminary study. *Indian Journal of Psychological Medicine*, *39*(4), 457–463. https://doi.org/10.4103/0253-7176.211767

Blader, J. C. (2004). Symptom, family, and service predictors of children's psychiatric rehospitalization within one year of discharge. *Journal of the American Academy of Child & Adolescent Psychiatry*, *43*(4), 440–451. https://doi.org/10.1097/00004583-200404000-00010

Bloom, R. B., & Hopewell, L. R. (1982). Psychiatric hospitalization of adolescents and successful mainstream reentry. *Exceptional Children*, *48*(4), 352–357. https://doi.org/10.1177/001440298204800410

Bobier, C., & Warwick, M. (2005). Factors associated with readmission to adolescent psychiatric care. *Australian & New Zealand Journal of Psychiatry*, *39*(7), 600–606. https://doi.org/10.1080/j.1440-1614.2005.01632.x

Brown, S.-A., Moeketsi, T. D., Musekiwa, A., & Moodley, S. V. (2023). Readmission of adolescent psychiatric patients to a specialised unit in Gauteng, South Africa. *South African Journal of Psychiatry*, *29*. https://doi.org/10.4102/sajpsychiatry.v29i0.2018

Carroll, A. R., Hall, M., Brown, C. M., Johnson, D. P., Antoon, J. W., Kreth, H., Ngo, M.-L., Browning, W., Neeley, M., Herndon, A., Chokshi, S. B., Plemmons, G., Johnson, J.,

Hart, S. R., & Williams, D. J. (2022). Association of race/ethnicity and social determinants with rehospitalization for mental health conditions at acute care children's hospitals. *The Journal of Pediatrics*, *240*, 228-234.e1. https://doi.org/10.1016/j.jpeds.2021.08.078

Charlemagne, S. J. (2012). *Family involvement, clinician beliefs and child psychiatric rehospitalization* [Dissertation, Loma Linda University]. https://www.proquest.com/docview/897969864/abstract/DF72353DB2D04AF2PQ/1

Chung, W., Edgar-Smith, S., Palmer, R. B., Bartholomew, E., & Delambo, D. (2008). Psychiatric rehospitalization of children and adolescents: Implications for social work intervention. *Child and Adolescent Social Work Journal*, *25*(6), 483–496. https://doi.org/10.1007/s10560-008-0147-8

Connell, S. K., Burkhart, Q., Tolpadi, A., Parast, L., Gidengil, C. A., Yung, S., Basco, W. T., Williams, D., Britto, M. T., Brittan, M., Wood, K. E., Bardach, N., McGalliard, J., & Mangione-Smith, R. (2021). Quality of care for youth hospitalized for suicidal ideation and self-harm. *Academic Pediatrics*, *21*(7), 1179–1186. https://doi.org/10.1016/j.acap.2021.05.019

Connell, S. K., To, T., Arora, K., Ramos, J., Haviland, M. J., & Desai, A. D. (2021). Perspectives of parents and providers on reasons for mental health readmissions: A content analysis study. *Administration and Policy in Mental Health and Mental Health Services Research*, *48*(5), 830–838. https://doi.org/10.1007/s10488-021-01134-6

Cotton, T. L. (2025). *Standardized transitional process in adolescent behavioral health: Evidence-based practice project* [Dissertation]. Liberty University.

Czyz, E. K., Berona, J., & King, C. A. (2016). Rehospitalization of suicidal adolescents in relation to course of suicidal ideation and future suicide attempts. *Psychiatric Services*, *67*(3), 332–338. https://doi.org/10.1176/appi.ps.201400252

Dahmen, B., Zielinski-Gussen, I., Föcker, M., Hahn, F., Legenbauer, T., Thiemann, U., Dempfle, A., & Herpertz-Dahlmann, B. (2024). Anorexia nervosa-specific home treatment in children and adolescents and their families (the HoT study): A study protocol of a randomized, controlled, multicenter, open-label, parallel group superiority trial. *Trials*, *25*(1), 760. https://doi.org/10.1186/s13063-024-08566-z

D'Aiello, B., Menghini, D., Averna, R., Labonia, M., & Vicari, S. (2021). Risk factors for revolving door in children and adolescents with psychiatric disorders. *Journal of Clinical Medicine*, *10*(21), 5004. https://doi.org/10.3390/jcm10215004

Day, D., McCullough, S., Scardamalia, K., Hunter, M., & Edwards, S. (2025). Patient-level and hospital treatment-level characteristics predicting child readmissions after psychiatric inpatient treatment. *The Journal of Behavioral Health Services & Research*, *52*(2), 330–341. https://doi.org/10.1007/s11414-024-09915-1

Deolmi, M., Turco, E. C., Pellegrini, P., Marchesi, C., & Pisani, F. (2021). Psychiatric emergency in children and adolescents: A retrospective study in parma local health unit. *Behavioural Neurology*, *2021*, e8848387. https://doi.org/10.1155/2021/8848387

Edgcomb, J. B., Sorter, M., Lorberg, B., & Zima, B. T. (2020). Psychiatric readmission of children and adolescents: A systematic review and meta-analysis. *Psychiatric Services*, *71*(3), 269–279. https://doi.org/10.1176/appi.ps.201900234

Eichstadt, S. A., Chetty, S., Magagula, T. G., & Swart, X. (2023). Factors affecting readmission of adolescent mental healthcare users to a psychiatric hospital. *South African Journal of Psychiatry*, *29*(0), Article 0. https://doi.org/10.4102/sajpsychiatry.v29i0.2110

Ekwemalor, C. C., Rozmus, C. L., Engebretson, J. C., Marcus, M. T., Casarez, R. L., & Harper, A. R. (2017). Treatment recidivism in adolescents with mental illness: A focused applied medical ethnography. *Journal of Child and Adolescent Psychiatric Nursing*, *30*(1), 25–34. https://doi.org/10.1111/jcap.12167

Falcone, T. (2021). *PASS (Promote Access to Stop Suicide): A Randomized Controlled Trial Comparing Enhanced Treatment as Usual, Crisis Center Follow up and Wraparound Services for Youth at Risk for Suicide* (Clinical Trial Registration No. NCT03016572). clinicaltrials.gov. https://clinicaltrials.gov/study/NCT03016572

Feng, J. Y., Toomey, S. L., Zaslavsky, A. M., Nakamura, M. M., & Schuster, M. A. (2017). Readmission after pediatric mental health admissions. *Pediatrics*, *140*(6), e20171571. https://doi.org/10.1542/peds.2017-1571

Fite, P. J., Stoppelbein, L., & Greening, L. (2009). Predicting readmission to a child psychiatric inpatient unit: The impact of parenting styles. *Journal of Child and Family Studies*, *18*(5), 621–629. https://doi.org/10.1007/s10826-009-9284-8

Fontanella, C. A. (2008). The influence of clinical, treatment, and healthcare system characteristics on psychiatric readmission of adolescents. *The American Journal of Orthopsychiatry*, *78*(2), 187–198. https://doi.org/10.1037/a0012557

Fontanella, C. A., Pottick, K. J., Warner, L. A., & Campo, J. V. (2010). Effects of medication management and discharge planning on early readmission of psychiatrically hospitalized adolescents. *Social Work in Mental Health*, *8*(2), 117–133. https://doi.org/10.1080/15332980902958149

Foster, E. M. (1999). Do aftercare services reduce inpatient psychiatric readmissions? *Health Services Research*, *34*(3), 715–736.

Franklin, E. V., Mathias, M., Nguyen, D. T., Hergenroeder, A. C., & Wiemann, C. M. (2025). A family navigator improves post-discharge treatment adherence among adolescents with anorexia nervosa: A pilot study. *Journal of Eating Disorders*, *13*(1), 109.

Gao, X., McSwiney, P., Court, A., Wiggins, A., & Sawyer, S. M. (2018). Somatic symptom disorders in adolescent inpatients. *Journal of Adolescent Health*, *63*(6), 779–784. https://doi.org/10.1016/j.jadohealth.2018.06.026

Gearing, R. E., Mian, I., Sholonsky, A., Barber, J., Nicholas, D., Lewis, R., Solomon, L., Williams, C., Lightbody, S., Steele, M., Davidson, B., Manchanda, R., Joseph, L., Handelman, K., & Ickowicz, A. (2009). Developing a risk-model of time to first-relapse for children and adolescents with a psychotic disorder. *The Journal of Nervous and Mental Disease*, *197*(1), 6. https://doi.org/10.1097/NMD.0b013e31819251d8

Grudnikoff, E., McNeilly, T., & Babiss, F. (2019). Correlates of psychiatric inpatient readmissions of children and adolescents with mental disorders. *Psychiatry Research*, *282*, 112596. https://doi.org/10.1016/j.psychres.2019.112596

Herrmann, E., Johns, H., Giles, E. M., McQuilton, P., Astbury, S., Matthews, R. V., & Lacey, J. H. (2025). To predict the prognosis of adolescents with anorexia nervosa leaving inpatient treatment: The development and initial evaluation of a novel tool to be used by a multi-disciplinary team. *Eating and Weight Disorders - Studies on Anorexia, Bulimia and Obesity*, *30*(1), 22. https://doi.org/10.1007/s40519-025-01731-6

Hillebrand, M. M. (1991). *A phenomenological investigation of adolescent girls' experience of psychiatric re-hospitalization* [Dissertation, The Union Institute]. https://www.proquest.com/docview/304031533/abstract/E387792A90E54704PQ/1

Hughes, E. K., Le Grange, D., Court, A., Yeo, M., Campbell, S., Whitelaw, M., Atkins, L., & Sawyer, S. M. (2014). Implementation of family-based treatment for adolescents with anorexia nervosa. *Journal of Pediatric Health Care*, *28*(4), 322–330. https://doi.org/10.1016/j.pedhc.2013.07.012

January, S.-A. A., Trout, A. L., Huscroft-D'Angelo, J., Duppong Hurley, K. L., & Thompson, R. W. (2018). Perspectives on factors impacting youth's reentry into residential care: An exploratory study. *Journal of Child and Family Studies*, *27*(8), 2584–2595. https://doi.org/10.1007/s10826-018-1093-5

Kagabo, R. (2016). *Exploring predictors of readmission for pediatric psychiatric inpatients* [Dissertation, University of Utah]. https://collections.lib.utah.edu/details?id=1426434

King, C. A., Hovey, J. D., Brand, E., & Ghaziuddin, N. (1997). Prediction of positive outcomes for adolescent psychiatric inpatients. *Journal of the American Academy of Child & Adolescent Psychiatry*, *36*(10), 1434–1442. https://doi.org/10.1097/00004583-199710000-00026

Lakin, B. L., Brambila, A. D., & Sigda, K. B. (2004). Parental involvement as a factor in the readmission to a residential treatment center. *Residential Treatment For Children & Youth*, *22*(2), 37–52. https://doi.org/10.1300/J007v22n02_03

Lammers, E. J., Zickafoose, J. S., Peterson, G. G., Blue, L., Stewart, K. A., & Kranker, K. (2019). Parent partners: Evaluation of a novel peer-support intervention for the caregivers of children hospitalized for behavioral health conditions. *Academic Pediatrics*, *19*(8), 908–916. https://doi.org/10.1016/j.acap.2019.06.001

Lapointe, A. R., Garcia, C., Taubert, A. L., & Sleet, M. G. (2010). Frequent use of psychiatric hospitalization for low-income, inner-city ethnic minority youth. *Psychological Services*, *7*(3), 162–176. https://doi.org/10.1037/a0019923

Lavanier, S. A. (2009). *The relationship between family functioning and readmissions of children on a psychiatric inpatient unit* [Dissertation, Xavier University]. https://www.proquest.com/docview/1430465610/abstract/D29D44177E3942DCPQ/1

Li, A., Cunich, M., Miskovic-Wheatley, J., Maloney, D., Madden, S., Wallis, A., & Maguire, S. (2021). Factors related to length of stay, referral on discharge and hospital readmission for children and adolescents with anorexia nervosa. *International Journal of Eating Disorders*, *54*(3), 409–421. https://doi.org/10.1002/eat.23399

Lohr, W. D., Jawad, K. S., Wood, N., Le, J. F., Stevenson, M. D., Feygin, Y. B., & Davis, D. W. (2024). Factors associated with child and youth mental health readmissions from a US national database. *JAACAP Open*, *2*(3), 170–179.

Lui, J. H., Chen, B. C., Benson, L. A., Lin, Y.-J. R., Ruiz, A., & Lau, A. S. (2024). Inpatient care utilization following mobile crisis response encounters among racial/ethnic

minoritized youth. *Journal of the American Academy of Child & Adolescent Psychiatry*, *63*(7), 720–732.

Markota, M., McKean, A. J., Romanowicz, M., Schak, K. M., Croarkin, P. E., & Vande Voort, J. L. (2018). Rehospitalization to a child and adolescent psychiatry unit: Role of trauma and bullying. *General Hospital Psychiatry*, *55*, 10–14. https://doi.org/10.1016/j.genhosppsych.2018.08.010

Mendes, P., Fonseca, M., Aguiar, I., Pangaio, N., Confraria, L., Queirós, O., Saraiva, J., Monteiro, P., & Guerra, J. (2017). Readmission to an adolescent psychiatry inpatient unit: Readmission rates and risk factors. *Acta Médica Portuguesa*, *30*(11), 769–774. https://doi.org/10.20344/amp.8842

Miller, D. A. A., Ronis, S. T., Slaunwhite, A. K., Audas, R., Richard, J., Tilleczek, K., & Zhang, M. (2020). Longitudinal examination of youth readmission to mental health inpatient units. *Child and Adolescent Mental Health*, *25*(4), 238–248. https://doi.org/10.1111/camh.12371

Milliren, C. E., Crowley, M., Carmody, J. K., Bern, E. M., Eldredge, O., & Richmond, T. K. (2024). Pediatric hospital utilization for patients with avoidant restrictive food intake disorder. *Journal of Eating Disorders*, *12*(1), 42. https://doi.org/10.1186/s40337-024-00996-z

Minchew, G. T. (2006). *Factors contributing to psychiatric rehospitalization and their application to adolescent males* [Dissertation, Capella University]. https://www.proquest.com/docview/304909736/abstract/1702C68E95C34029PQ/1

Mumbere Vagheni, M., Mutume Nzanzu Vivalya, B., Kasereka Muyisa, L., Kasereka Masuka, R., & Manzekele Bin Kitoko, G. (2022). Prevalence and predictors of relapse among adolescent patients with mental illness in Butembo city (Eastern Part of the Democratic Republic of Congo). *Psychiatry Research*, *308*, 114342. https://doi.org/10.1016/j.psychres.2021.114342

Neumann, A., Hense, H., Baum, F., Kliemt, R., Seifert, M., Harst, L., Kubat, D., Maicher, B., Schrey, C., Schmitt, J., Pfennig, A., Weinhold, I., Swart, E., & Soltmann, B. (2021). Evaluation of a flexible and integrative psychiatric care model in a department of child and adolescent psychiatry in Tübingen, Germany: Study protocol (EVA_TIBAS). *BMC Health Services Research*, *21*(1), 1262. https://doi.org/10.1186/s12913-021-07226-1

Ney, P. G., & Mills, W. A. (1976). A time-limited treatment program for children and their families. *Psychiatric Services*, *27*(12), 878–879. https://doi.org/10.1176/ps.27.12.878

Oltean, I. I., Perlman, C., Meyer, S., & Ferro, M. A. (2020). Child mental illness and mental health service use: Role of family functioning (family functioning and child mental health). *Journal of Child and Family Studies*, *29*(9), 2602–2613. https://doi.org/10.1007/s10826-020-01784-4

Otterson, S. E., Fristad, M. A., McBee-Strayer, S., Bruns, E., Chen, J., Schellhause, Z., Bridge, J., & Murphy, M. A. (2021). Length of stay and readmission data for adolescents psychiatrically treated on a youth crisis stabilization unit versus a traditional inpatient unit. *Evidence-Based Practice in Child and Adolescent Mental Health*, *6*(4), 484–489. https://doi.org/10.1080/23794925.2021.1986868

Pavkov, T. W., Goerge, R. M., & Lee, B. J. (1997). State hospital reentry among youth with serious emotional disturbance: A longitudinal analysis. *Journal of Child and Family Studies*, *6*(3), 373–383. https://doi.org/10.1023/A:1025008404511

Persi, J., & Sisson, M. (2008). Children in foster care: Before, during, and after psychiatric hospitalization. *Child Welfare*, *87*(4), 79–99.

Phillips, M. S., Steelesmith, D. L., Campo, J. V., Pradhan, T., & Fontanella, C. A. (2020). Factors associated with multiple psychiatric readmissions for youth with mood disorders. *Journal of the American Academy of Child & Adolescent Psychiatry*, *59*(5), 619–631. https://doi.org/10.1016/j.jaac.2019.05.024

Pieterse, D., Temmingh, H., & Vogel, W. (2016). Factors associated with readmission in South African adolescents discharged from two inpatient psychosocial rehabilitation units. *Journal of Child & Adolescent Mental Health*, *28*(3), 199–212. https://doi.org/10.2989/17280583.2016.1259165

Ramsbottom, H., & Farmer, L. C. (2018). Reducing pediatric psychiatric hospital readmissions and improving quality care through an innovative Readmission Risk Predictor Tool. *Journal of Child and Adolescent Psychiatric Nursing*, *31*(1), 14–22. https://doi.org/10.1111/jcap.12203

Rhodes, P., & Madden, S. (2005). Scientist-practitioner family therapists, postmodern medical practitioners and expert parents: Second-order change in the Eating Disorders Program at The Children's Hospital at Westmead. *Journal of Family Therapy*, *27*(2), 171–182. https://doi.org/10.1111/j.1467-6427.2005.00309.x

Sams, D. P., Garrison, D., Walsh, P., Maeng, D., & Cross, W. (2023). The rapid stabilization pathway: Impact of a brief inpatient intervention on the length of stay and readmissions among psychiatrically hospitalized adolescents. *Child and Adolescent Mental Health*, *28*(4), 481–487. https://doi.org/10.1111/camh.12636

Seleem, M. A., & Amer, R. A. (2021). Demographic & clinical correlates of admission into a specialized psychiatric inpatient service for children and adolescents in Egypt: An observational retrospective study. *Journal of Psychiatric and Mental Health Nursing*, *28*(6), 970–980. https://doi.org/10.1111/jpm.12730

Sheifer, M., Pavlova, M., Ashcheulova, T., Chigareva, O., Bikbaeva, K., Smirnova, D., & Syunyakov, T. (2024). Self-harm behavior and suicidality among adolescents in Samara region. *Psychiatria Danubina*, *36*, 218–224.

Similä, N., Hakko, H., Riipinen, P., & Riala, K. (2018). Gender specific characteristics of revolving door adolescents in acute psychiatric inpatient care. *Child Psychiatry & Human Development*, *49*(2), 225–233. https://doi.org/10.1007/s10578-017-0744-x

Simmons, S., Noble, C., Arquette, K., & Thompson, A. D. (2022). Bridging the gap: Impact of dedicated post-discharge services on readmission rates and emergency department visits for psychiatrically hospitalized youth. *Clinical Child Psychology and Psychiatry*, 13591045221125634. https://doi.org/10.1177/13591045221125634

Solerdelcoll, M., Ilzarbe, D., Fortea, A., Morer, A., Lazaro, L., Sugranyes, G., & Baeza, I. (2024). Psychopathology and mental health service use among youth in foster care admitted to a psychiatric inpatient unit: A 4-year retrospective controlled study. *European Child & Adolescent Psychiatry*, *33*(1), 39–50. https://doi.org/10.1007/s00787-022-02104-5

Soutullo, O. R., Duncan, F., Coleman, L., Mitchell, S. J., Godoy, L., Tyson, C., & Long, M. (2023). Factors associated with psychiatric readmission of youths in a racially diverse and urban hospital setting. *Psychiatric Services*, *74*(1), 66–69. https://doi.org/10.1176/appi.ps.202100389

Steinhausen, H.-C., Grigoroiu-Serbanescu, M., Boyadjieva, S., Neumärker, K.-J., & Winkler Metzke, C. (2008). Course and predictors of rehospitalization in adolescent anorexia nervosa in a multisite study. *International Journal of Eating Disorders*, *41*(1), 29–36. https://doi.org/10.1002/eat.20414

Stewart, S. L., Kam, C., & Baiden, P. (2014). Predicting length of stay and readmission for psychiatric inpatient youth admitted to adult mental health beds in Ontario, Canada. *Child and Adolescent Mental Health*, *19*(2), 115–121. https://doi.org/10.1111/camh.12022

Stoner, A. M. (2014). *Closing the "revolving door": Identifying predictors of time to rehospitalization in a sample of psychiatric inpatient youth* [Dissertation, Loyola University Chicago]. https://www.proquest.com/docview/1622149745/abstract/311753BB892A43ADPQ/1

Vadén, V., Kaltiala, R., & Holttinen, T. (2024). Readmissions to adolescent psychiatric inpatient care: A register study. *Psychiatria Fennica*, *55*, 186–197.

van Alphen, N. R., Stewart, J. G., Esposito, E. C., Pridgen, B., Gold, J., & Auerbach, R. P. (2017). Predictors of rehospitalization for depressed adolescents admitted to acute psychiatric treatment. *The Journal of Clinical Psychiatry*, *78*(5), 592–598. https://doi.org/10.4088/JCP.15m10326

Wallin, U., & Holmer, R. (2021). Long-term outcome of adolescent anorexia nervosa: Family treatment apartments compared with child psychiatric inpatient treatment. *Frontiers in Psychiatry*, *12*. https://doi.org/10.3389/fpsyt.2021.640622

Wallis, A., Rhodes, P., Kohn, M., & Madden, S. (2007). Five-years of family based treatment for anorexia nervosa: The Maudsley Model at the Children's Hospital at Westmead. *International Journal of Adolescent Medicine and Health*, *19*(3), 277–284. https://doi.org/10.1515/IJAMH.2007.19.3.277

Wang, H., Xie, X., Li, Z., Xue, L., & Liao, Q. (2024). Perceived family functioning of adolescents with affective disorders: A multicentric cross-sectional study. *Psychology Research and Behavior Management*, *17*, 2505–2517. https://doi.org/10.2147/PRBM.S460961

Ward, J. L., Vázquez-Vázquez, A., Phillips, K., Settle, K., Pilvar, H., Cornaglia, F., Gibson, F., Nicholls, D., Roland, D., Mathews, G., Roberts, H., Viner, R. M., & Hudson, L. D. (2025). Admission to acute medical wards for mental health concerns among children and young people in England from 2012 to 2022: A cohort study. *The Lancet Child & Adolescent Health*, *9*(2), 112–120. https://doi.org/10.1016/S2352-4642(24)00333-X

Winner, K., Sandberg, K., Saia, B., Calabro, M., & Blankenship, K. (2025). A Quality improvement initiative to decrease behavioral health unit readmission rates. *Pediatrics*, *155*(2), e2023064917.

Yampolskaya, S., Mowery, D., & Dollard, N. (2013). Predictors for readmission into children's inpatient mental health treatment. *Community Mental Health Journal*, *49*(6), 781–786. https://doi.org/10.1007/s10597-013-9592-8

Yampolskaya, S., Mowery, D., & Dollard, N. (2014). Profile of children placed in residential psychiatric program: Association with delinquency, involuntary mental health commitment, and reentry into care. *American Journal of Orthopsychiatry*, *84*(3), 234–243. https://doi.org/10.1037/h0099808

Yen, S., Fuller, A., Joel, S., & Spirito, A. (2014). Follow-up treatment utilization by hospitalized suicidal adolescents. *Journal of Psychiatric Practice*, *20*(5), 353–362. https://doi.org/10.1097/01.pra.0000454780.59859.9e

Yen, S., Spirito, A., Weinstock, L. M., Tezanos, K., Kolobaric, A., & Miller, I. (2019). Coping long term with active suicide in adolescents: Results from a pilot randomized controlled trial. *Clinical Child Psychology and Psychiatry*, *24*(4), 847–859. https://doi.org/10.1177/1359104519843956

Yu, A. G., Hall, M., Agharokh, L., Lee, B. C., Zaniletti, I., Wilson, K. M., & Williams, D. J. (2022). Hospital-level neighborhood opportunity and rehospitalization for common diagnoses at US children's hospitals. *Academic Pediatrics*, *22*(8), 1459–1467. https://doi.org/10.1016/j.acap.2022.05.008
