# Supplementary material for: The role of the family in youth psychiatric readmission: a scoping review
Source: Eur Child Adolesc Psychiatry. 2026 Jun 6;35(8):2349–65. doi: 10.1007/s00787-026-03062-y (PMC13652881; doi:10.1007/s00787-026-03062-y)
Supplement: Supplementary file 2 [file 787_2026_3062_MOESM2_ESM.pdf]

**Online Resource 1**

**Study selection and data extraction**

Corresponding author: Amos-Silvio E. Friedrich, University of Vienna

(amos.friedrich@univie.ac.at)

## Selection Process (Detailed)

Screening was piloted by the first and third author (AF, JB) using a random set of 200 records (92% agreement, Brennan-Prediger κ = .84), clarifying eligibility criteria and repeating the screening process on a new random sample of 500 records (98% agreement, κ = .96). The same authors then independently screened titles and abstracts of all records of the initial search according to the specified eligibility criteria. Disagreements during screening were resolved by consensus across authors (AF, JB, MZ). Final eligibility was determined based on a full text assessment, again conducted independently by the same two coders (99.5% agreement, κ = .98). Disagreements on fulltext eligibility were resolved by consensus or consulting the senior author (MZ).

The search updates were conducted by the first (AF) and fourth (MR) author. Titles and abstracts were screened independently for the first ($k$ = 597, 98% agreement, κ = .96) and second update ($k$ = 683, 95% agreement, κ = .89) and final eligibility was determined after independent assessment of full texts. Screening and full text disagreements were resolved by consensus (AF, MR, MZ).

**Data Items (detailed)**

For each study, we extracted the following information (see also our coding sheet on OSF):

- Publication and study characteristics: Year of publication, author(s), country or countries of data collection, study design (qualitative, quantitative, mixed methods, review), study perspective (prospective/retrospective), mode(s) of assessment (intervention, questionnaire, interview, observation, record analysis), source of data (patient, parent, clinician, researcher, administrative data)
- Sample characteristics: Population (sampling frame) of index patients, sample size of index patients, population (sampling frame) of family members, sample size of family members, if intervention: population (sampling frame) of control group(s), sample size of control group(s), age of index patients (*M, SD*), gender composition of index patients (mixed, boys only, girls only), year(s) of data collection
- Conceptual information: Study aim(s) or research questions (author reported), readmission measure (e.g., to same/any facility, timeframe), readmission rate (% readmitted), time to readmission (*M, SD*), nature of the intervention or assessed family-related variables/constructs, study findings regarding family contributions (author reported, from results and discussion sections), study limitations (author reported, from discussion sections), research recommendations (author reported, from discussion section)
