# Supplementary material for: The role of the family in youth psychiatric readmission: a scoping review
Source: Eur Child Adolesc Psychiatry. 2026 Jun 6;35(8):2349–65. doi: 10.1007/s00787-026-03062-y (PMC13652881; doi:10.1007/s00787-026-03062-y)
Supplement: Supplementary file 3 [file 787_2026_3062_MOESM3_ESM.pdf]

Online Resource 4

Methods and sources of evidence

Overview

Fig. S4.1. Methods and sources of evidence utilized in included studies

Fig. S4.2. Study perspectives over time

Corresponding author: Amos-Silvio E. Friedrich, University of Vienna (amos.friedrich@univie.ac.at)

Fig. S4.1 Methods and sources of evidence utilized in included studies

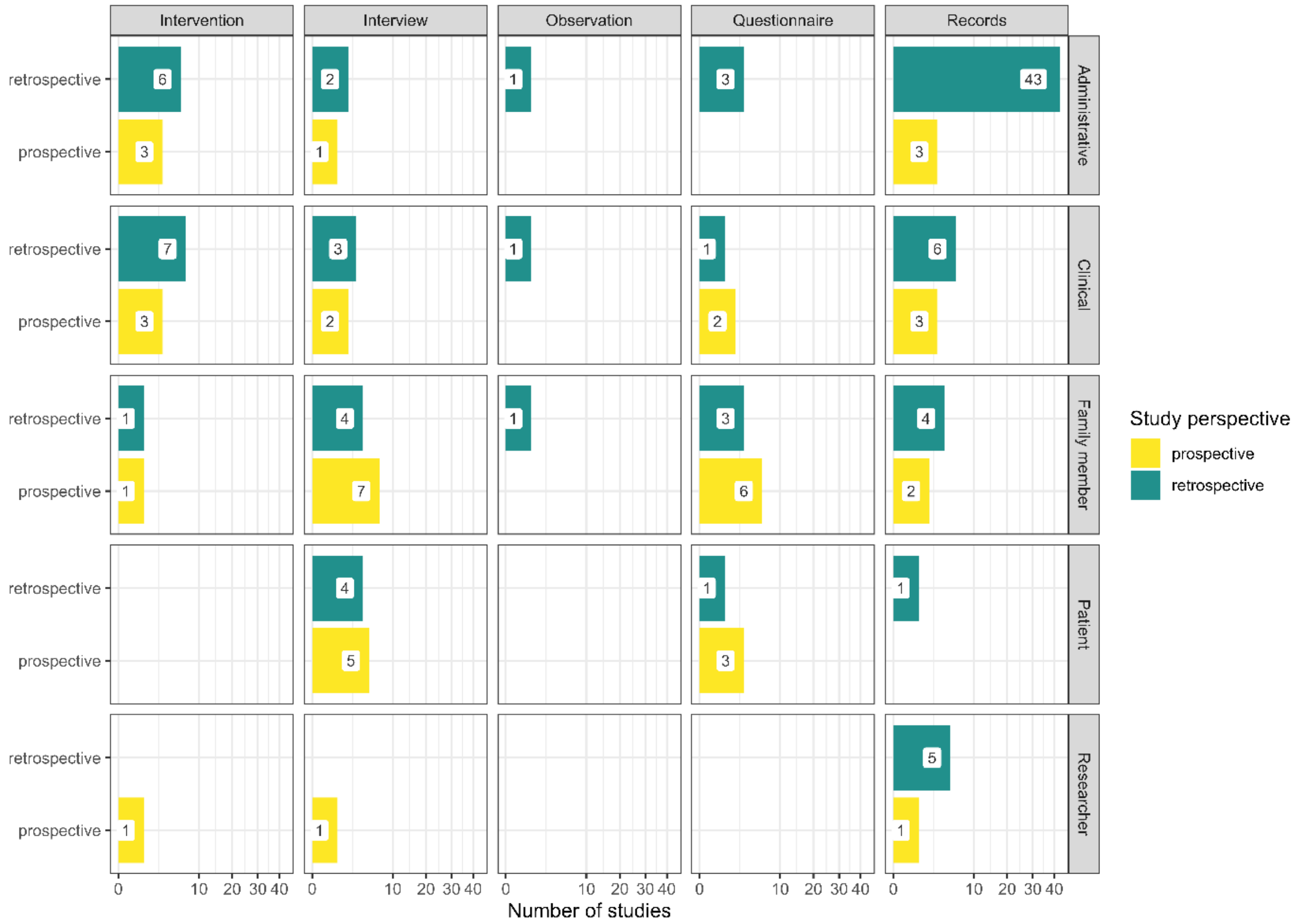

Note. Number of studies are shown separately by methods (horizontal) and sources of evidence (vertical) used to assess family variables. Axis scaling was square-root-transformed to improve the readability of low-count combinations. Studies utilizing multiple methods or sources were counted once for each combination, so the totals reported in the main text and those shown in this figure may differ.

Fig. S4.2 Study perspectives over time

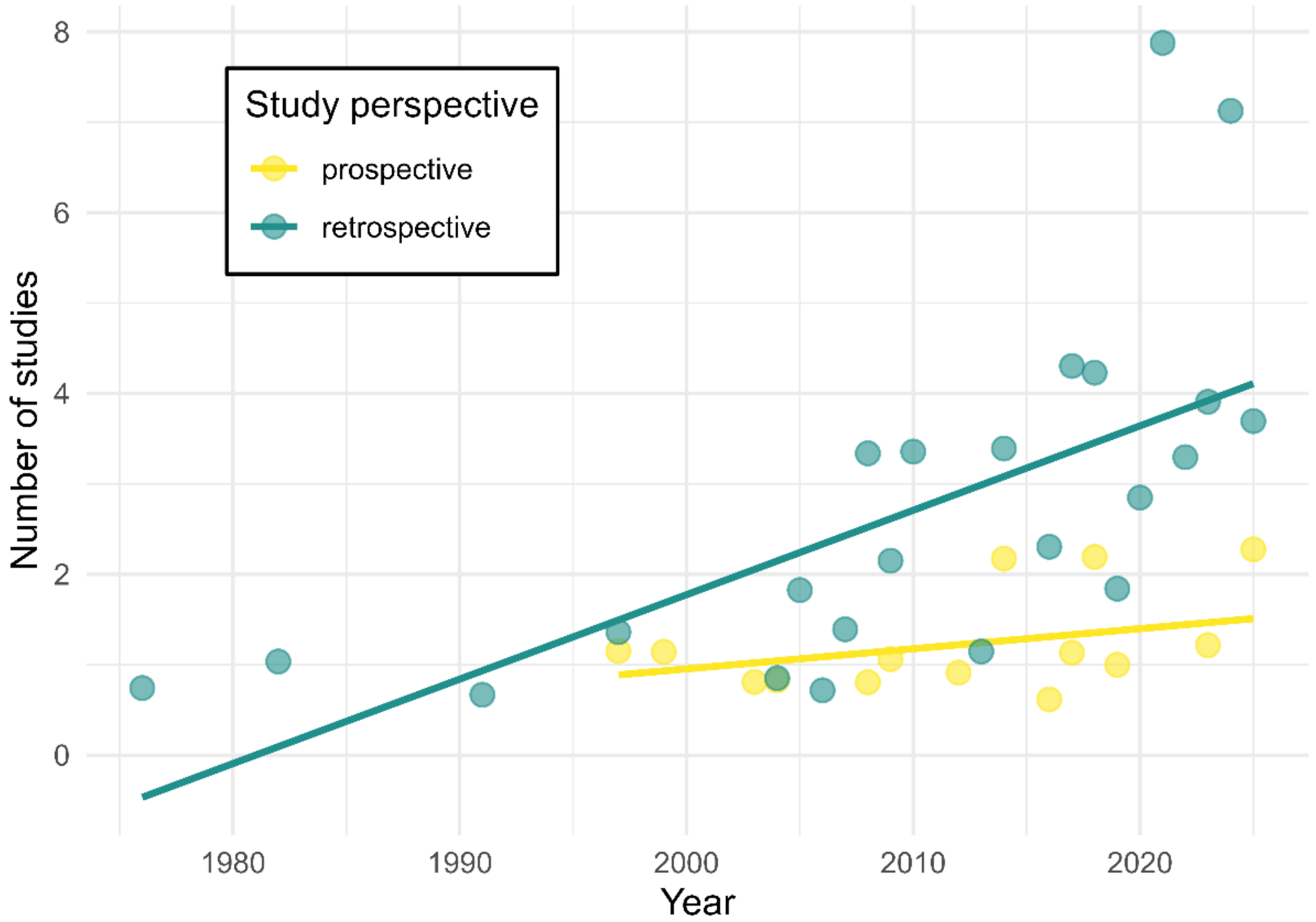

Note. Lines indicate linear trends. Points indicate number of publications per year and are slightly jittered on the y-axis to improve readability.
