# Supplementary material for: The role of the family in youth psychiatric readmission: a scoping review
Source: Eur Child Adolesc Psychiatry. 2026 Jun 6;35(8):2349–65. doi: 10.1007/s00787-026-03062-y (PMC13652881; doi:10.1007/s00787-026-03062-y)
Supplement: Supplementary file 4 [file 787_2026_3062_MOESM4_ESM.pdf]

Online Resource 5

Limitation and recommendation themes coded for included studies

Overview

Table S5.1. Limitation themes of included studies

Table S5.2. Recommendation themes of recent studies

Corresponding author: Amos-Silvio E. Friedrich, University of Vienna (amos.friedrich@univie.ac.at)

Table S5.1. Limitation themes of included studies

Limitation theme	IDs
Unexamined variables (k = 34)	1, 2, 8, 11, 20, 23, 25, 28, 29, 30, 31, 33, 37, 39, 40, 41, 43, 45, 49, 50, 52, 53, 54, 57, 58, 62, 63, 64, 65, 66, 72, 75, 76, 77
Biased sample (k = 25)	1, 4, 8, 12, 14, 19, 23, 24, 27, 28, 30, 34, 37, 41, 45, 52, 53, 57, 60, 62, 65, 68, 73, 75, 77
Sample size / power issues (k = 23)	2, 3, 6, 13, 19, 21, 25, 26, 29, 30, 32, 36, 38, 43, 47, 50, 58, 59, 60, 61, 64, 66, 78
Uncaptured readmissions (k = 21)	2, 7, 8, 11, 16, 18, 20, 22, 28, 32, 33, 38, 42, 44, 53, 54, 61, 63, 68, 73, 79
Single site (k = 19)	1, 4, 9, 10, 12, 16, 17, 18, 19, 29, 32, 36, 46, 55, 57, 63, 66, 67, 77
Potentially unreliable data source/measure (k = 18)	1, 3, 12, 16, 22, 26, 27, 33, 36, 38, 40, 47, 49, 55, 66, 72, 74, 76
Incomplete or inaccurate data (k = 15)	7, 22, 39, 41, 42, 44, 46, 54, 58, 60, 64, 67, 69, 77, 79
Limited timeframe (k = 15)	2, 4, 6, 7, 10, 13, 17, 18, 21, 25, 38, 63, 66, 75, 77
Retrospective (k = 15)	2, 3, 6, 14, 16, 25, 27, 47, 49, 50, 62, 67, 71, 72, 79
Observational/noncontrolled design (k = 12)	3, 14, 16, 21, 24, 31, 34, 35, 37, 53, 60, 79
Nonrandom allocation (k = 5)	25, 50, 61, 69, 71
Low response rate or attrition (k = 4)	9, 27, 28, 29
Proxy variables (k = 4)	8, 41, 63, 79
Outdated data (k = 3)	22, 23, 53
Unspecific intervention (k = 2)	61, 64

Note. Limitation themes were considered for all empirical studies (79 studies). One or more limitations were extracted from 73 of those studies.

Table S5.2. Recommendation themes of recent studies

Recommendation theme	IDs
Methods	
Large-scale data (k = 9)	19, 25, 32, 36, 47, 49, 50, 57, 66
Prospective designs (k = 6)	26, 47, 49, 50, 54, 62
Expand data sources (k = 5)	19, 26, 39, 66, 77
Longer timeframes (k = 4)	25, 36, 39, 75
Innovative methods (k = 3)	20, 75, 77
RCT (k = 2)	50, 62
Examine	
Follow-up care (k = 12)	13, 14, 20, 29, 31, 32, 40, 61, 63, 65, 66, 74
Social disparities (k = 11)	8, 11, 12, 14, 41, 45, 60, 63, 67, 72, 79
Family-focused care (k = 7)	29, 40, 50, 65, 69, 72, 73
Caregiver factors (k = 5)	25, 26, 28, 65, 66
Community factors (k = 5)	7, 18, 20, 40, 45
Post-discharge period (k = 4)	32, 33, 57, 63
Alternate modalities (k = 4)	33, 58, 65, 78
Comorbidity (k = 4)	7, 29, 54, 77
Causality (k = 2)	28, 41
Differential indication (k = 2)	31, 61
Experience (k = 2)	31, 57
Service access (k = 2)	32, 41
Develop	
Improve treatments (k = 8)	42, 44, 49, 59, 67, 71, 73, 77
Predictive tools/models (k = 6)	29, 47, 53, 55, 75, 79
Personalization (k = 2)	72, 78

Note. Recommendations were considered for all studies published after 2013 (54 studies).

One or more recommendation themes were extracted from 49 of those studies.
