# Supplementary material for: The role of the family in youth psychiatric readmission: a scoping review
Source: Eur Child Adolesc Psychiatry. 2026 Jun 6;35(8):2349–65. doi: 10.1007/s00787-026-03062-y (PMC13652881; doi:10.1007/s00787-026-03062-y)

Online Resource 3

Supplementary figures

Overview

Figure S3.1. Effect directions for complex subcategories

Figure S3.2. Bubble chart of bivariate associations

Figure S3.3. Bubble chart of multivariate associations

Corresponding author: Amos-Silvio E. Friedrich, University of Vienna

(amos.friedrich@univie.ac.at)

Figure S3.1 Effect directions for complex subcategories

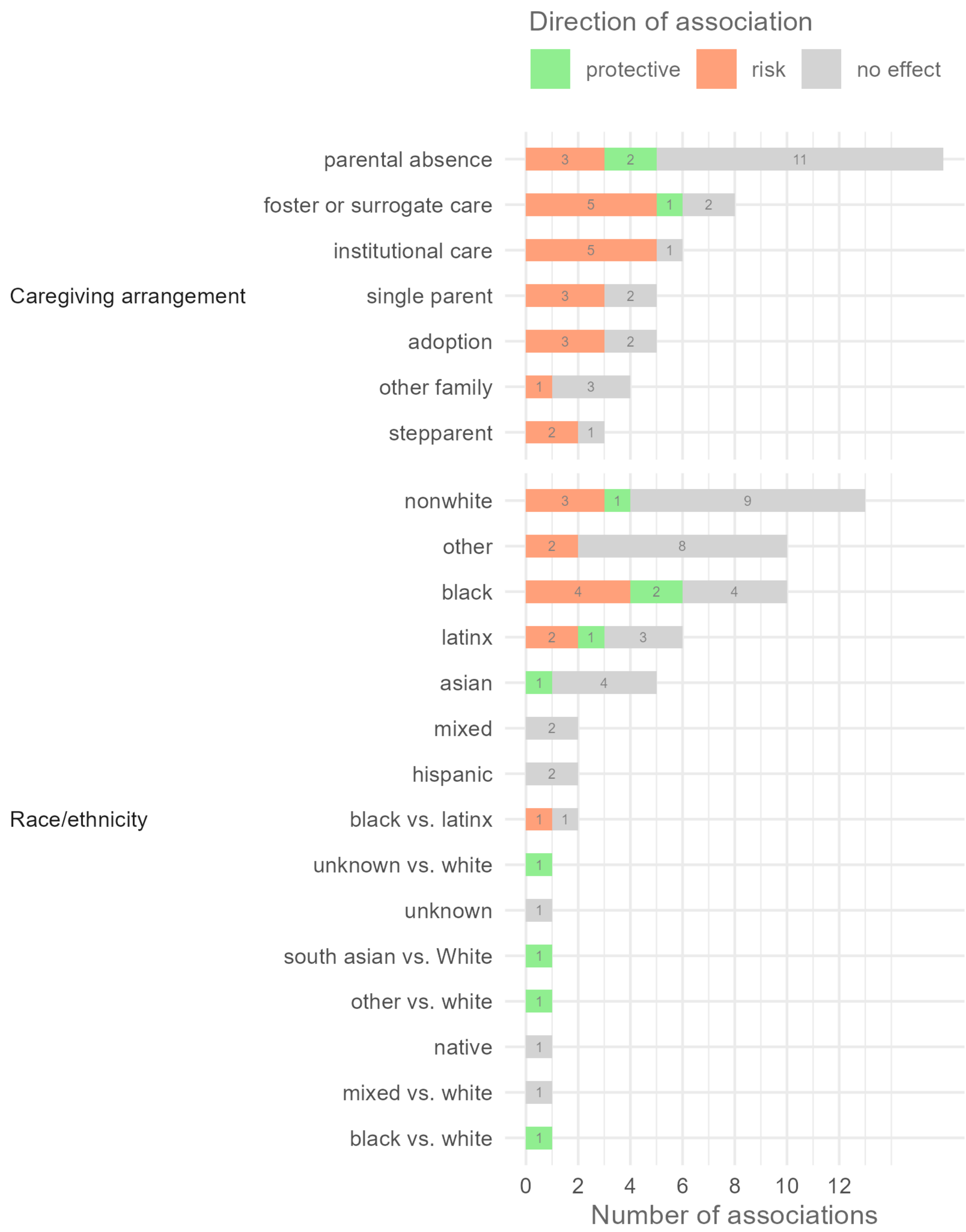

Note. Race/ethnicity categories without a specified comparison group were compared against white or all other subgroups.

Figure S3.2 Bubble chart of bivariate associations

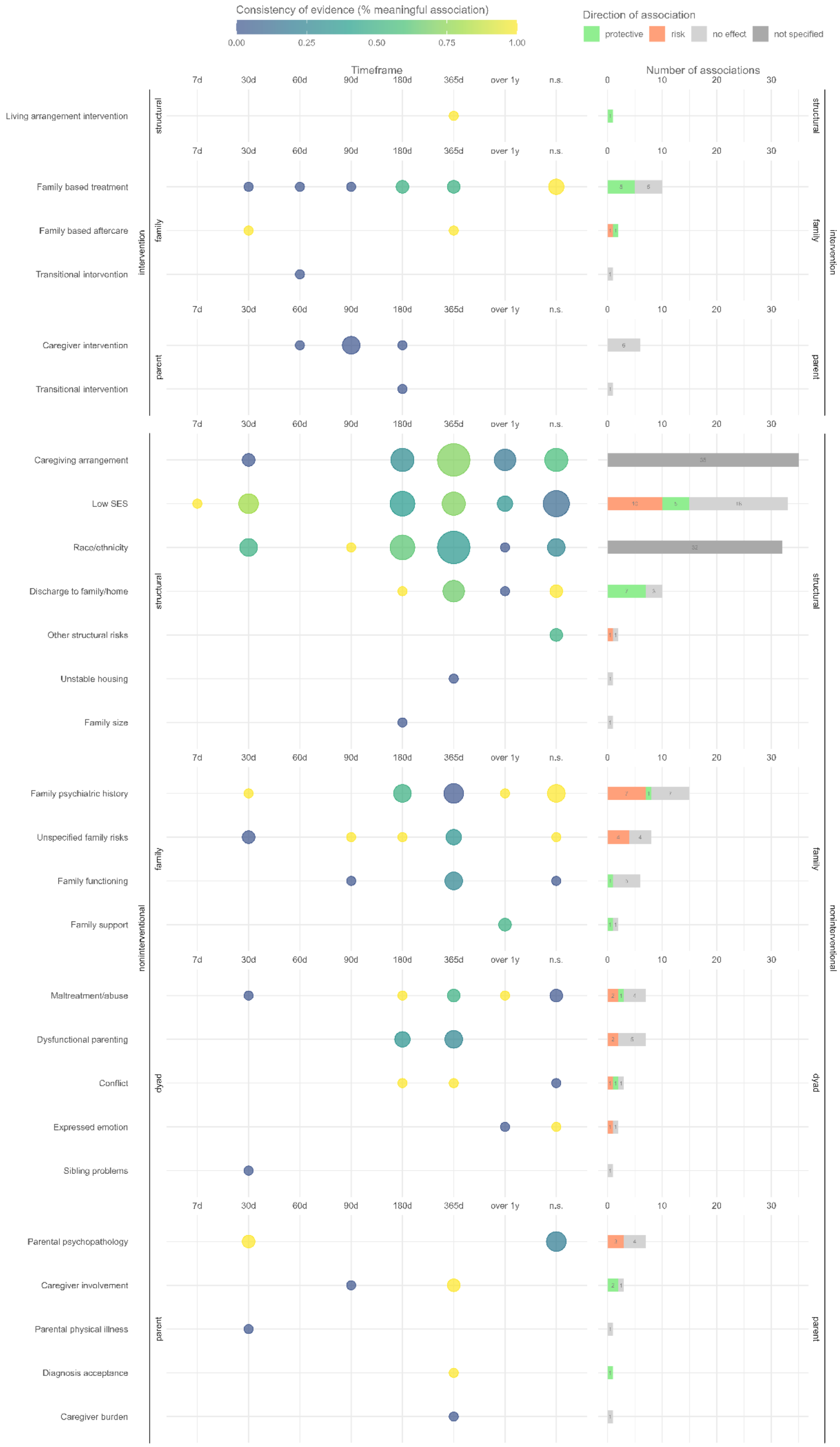

Figure S3.3 Bubble chart of multivariate associations

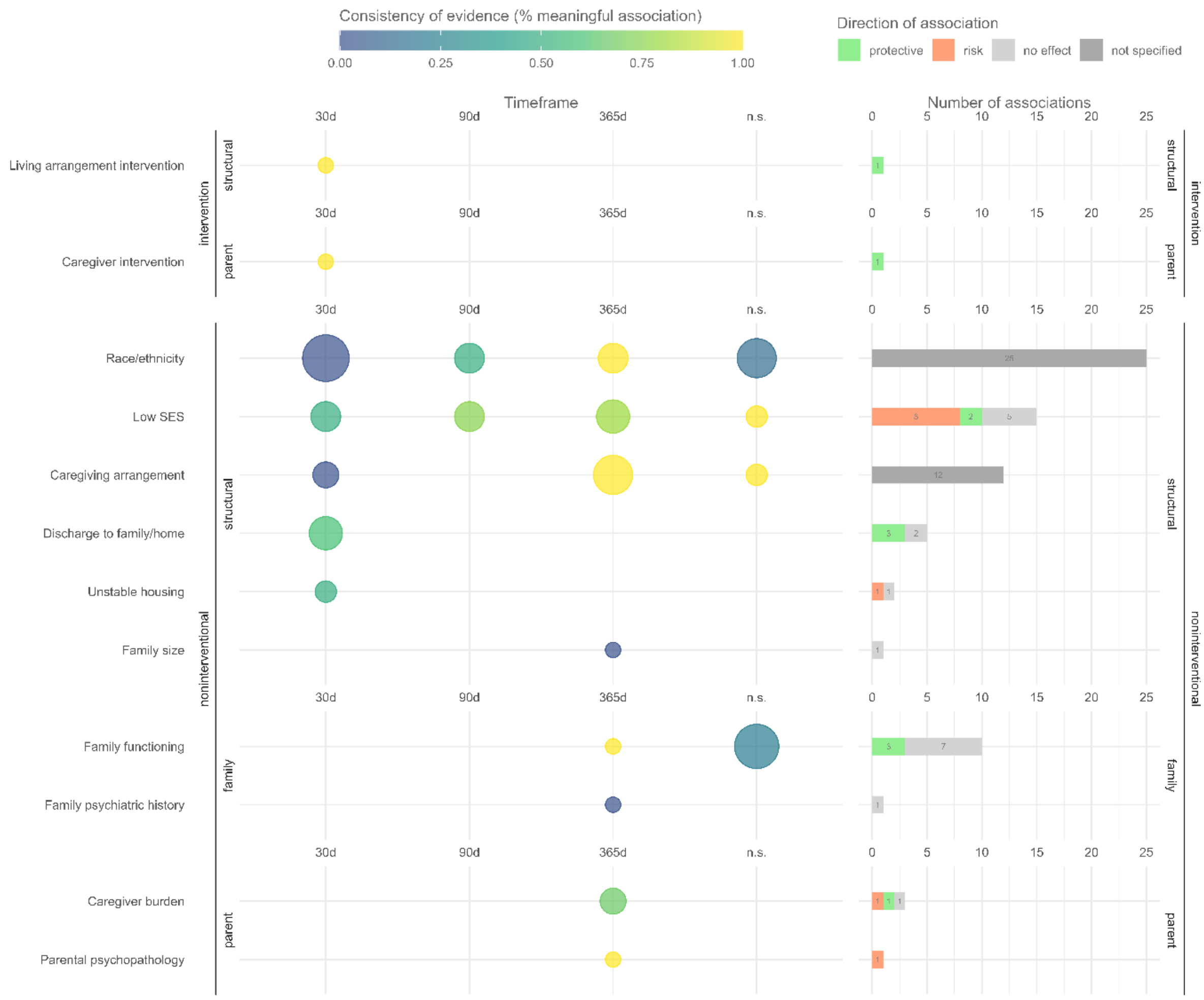

Supplement: Supplementary file 5 [file 787_2026_3062_MOESM5_ESM.pdf]
